# Immunohistochemistry and Mutation Analysis of *SDHx* Genes in Carotid Paragangliomas

**DOI:** 10.3390/ijms21186950

**Published:** 2020-09-22

**Authors:** Anastasiya V. Snezhkina, Dmitry V. Kalinin, Vladislav S. Pavlov, Elena N. Lukyanova, Alexander L. Golovyuk, Maria S. Fedorova, Elena A. Pudova, Maria V. Savvateeva, Oleg A. Stepanov, Andrey A. Poloznikov, Tatiana B. Demidova, Nataliya V. Melnikova, Alexey A. Dmitriev, George S. Krasnov, Anna V. Kudryavtseva

**Affiliations:** 1 Center for Precision Genome Editing and Genetic Technologies for Biomedicine, Engelhardt Institute of Molecular Biology, Russian Academy of Sciences, 119991 Moscow, Russia; vladislav1pavlov@gmail.com (V.S.P.); lukianovaelena@yandex.ru (E.N.L.); fedorowams@yandex.ru (M.S.F.); pudova_elena@inbox.ru (E.A.P.); msavv@mail.ru (M.V.S.); ollegstepanov@gmail.com (O.A.S.); mnv-4529264@yandex.ru (N.V.M.); alex_245@mail.ru (A.A.D.); gskrasnov@mail.ru (G.S.K.); 2 Vishnevsky Institute of Surgery, Ministry of Health of the Russian Federation, 117997 Moscow, Russia; dmitry.v.kalinin@gmail.com (D.V.K.); algolovyuk@inbox.ru (A.L.G.); 3 National Medical Research Radiological Center, Ministry of Health of the Russian Federation, 125284 Moscow, Russia; andrey.poloznikov@gmail.com; 4 A. N. Severtsov Institute of Ecology and Evolution, Russian Academy of Sciences, 119071 Moscow, Russia; demidovatanya@mail.ru

**Keywords:** carotid paraganglioma, *SDHx* genes, mutations, protein expression, exome sequencing, immunohistochemistry

## Abstract

Carotid paragangliomas (CPGLs) are rare neuroendocrine tumors often associated with mutations in *SDHx* genes. The immunohistochemistry of succinate dehydrogenase (SDH) subunits has been considered a useful instrument for the prediction of *SDHx* mutations in paragangliomas/pheochromocytomas. We compared the mutation status of *SDHx* genes with the immunohistochemical (IHC) staining of SDH subunits in CPGLs. To identify pathogenic/likely pathogenic variants in *SDHx* genes, exome sequencing data analysis among 42 CPGL patients was performed. IHC staining of SDH subunits was carried out for all CPGLs studied. We encountered *SDHx* variants in 38% (16/42) of the cases in *SDHx* genes. IHC showed negative (5/15) or weak diffuse (10/15) SDHB staining in most tumors with variants in any of *SDHx* (94%, 15/16). In *SDHA*-mutated CPGL, SDHA expression was completely absent and weak diffuse SDHB staining was detected. Positive immunoreactivity for all SDH subunits was found in one case with a variant in *SDHD*. Notably, CPGL samples without variants in *SDHx* also demonstrated negative (2/11) or weak diffuse (9/11) SDHB staining (42%, 11/26). Obtained results indicate that SDH immunohistochemistry does not fully reflect the presence of mutations in the genes; diagnostic effectiveness of this method was 71%. However, given the high sensitivity of SDHB immunohistochemistry, it could be used for initial identifications of patients potentially carrying *SDHx* mutations for recommendation of genetic testing.

## 1. Introduction

Carotid paraganglioma (CPGL) is a rare neuroendocrine tumor that arises from the carotid body. CPGL represents more than half of all head and neck (HN) paragangliomas (PGLs) [1]. According to the WHO Classification of Head and Neck Tumors 2017, PGLs were reclassified from indeterminate to malignant tumors with variable potential of metastasis [2]. As CPGL associates with the carotid arteries and adjacent nerves, its resection is challenging. Potential surgical complications include stroke, the possibility of cerebrovascular accident, and neural dysfunction, which can manifest clinically as hoarseness, vocal change, aspiration, dysphagia, dysarthria, facial asymmetry, or shoulder weakness [3]. Radiotherapy and chemotherapy can be used for unresectable and metastatic tumors, as well as for bilateral CPGLs; however, surgery remains the primary form of treatment [4,5,6].

CPGLs can develop both in the familial and sporadic forms [7]. Familial CPGLs occur as hereditary paraganglioma/pheochromocytoma (PGL/PCC) syndromes, including PGL1, PGL2, PGL3, PGL4, and PGL5, which are associated with germline mutations in the *SDHD*, *SDHAF2*, *SDHC*, *SDHB*, and *SDHA* genes, respectively [8]. *SDHx* genes encode for subunits of succinate dehydrogenase (SDH, mitochondrial complex II); SDHAF2 protein participates in the assembly of SDH and is required for SDHA flavination [9]. Two novel genes, *TMEM127* and *MAX*, were recently found contributive to hereditary PGL/PCC syndromes. Germline pathogenic variants in these genes are mainly associated with PCCs but have also been seen in CPGLs [10,11]. Apparently sporadic cases of CPGLs have been reported as being associated with germline mutations in *SDHA*, *SDHB*, *SDHD*, and *VHL*, as well as somatic mutations in *VHL*, *RET*, *IDH1*, and *IDH2* [12,13,14,15,16,17,18,19]. Germline pathogenic mutations in the *FH* and *SLC25A11* genes were also determined in CPGLs [20,21].

The immunohistochemistry (IHC) of SDHB and SDHA subunits has been proposed as a useful method to predict underlying *SDHx* mutations in first-line diagnostics of hereditary PCCs/PGLs. All PGLs carrying *SDHA* mutations showed negative SDHA staining, while SDHA-positive immunoreactivity was observed in tumors with mutations in other *SDHx* genes [12]. Loss of cytoplasmic SDHB staining or a weak diffuse cytoplasmic blush instead of a normal granular staining pattern was revealed in PGLs with mutations in *SDHB, SDHC,* or *SDHD* [22,23]. Additionally, several cases of PGLs/PCCs with *SDHA* mutations showed a loss of SDHB protein expression coupled with negative SDHA staining [24]. To validate the reproducibility of SDHA/SDHB immunohistochemistry for the identification of hereditary PGLs/PCCs, seven expert endocrine pathologists performed analyses of 351 tumors with known/unknown genetics using a web-based virtual microscopy approach [24]. Pathologists reached an agreement in 99% (348/351) of the interpretations of SDHA immunohistochemistry and 90% (315/351) of the cases for SDHB. About 90% of PGLs/PCCs with mutations in *SDHB, SDHC, SDHD,* and *SDHAF2* were SDHB immunonegative and SDHA immunopositive. Loss of SDHA/SDHB staining was detected in 75% of tumors with *SDHA* mutations. Finally, SDHA/SDHB protein expression was positive in 93% of non-*SDHx*-mutated cases. These results established the usability of SDHA/SDHB immunohistochemistry to identify *SDHx* mutations in PGLs/PCCs. Nevertheless, in some cases, the IHC results may be misinterpreted or reflect the wrong mutation status. For example, changed SDHB immunostaining can be caused by not only exonic mutations in the *SDHx* genes but also by the epigenetic alterations (epimutations), mutations in regulatory regions, changes in the expression and functionality of transcription factors, or other components of energy metabolism [23].

Another method for prediction of *SDHx* mutation status bases on evaluating the succinate-to-fumarate ratio (SFR) using liquid chromatography–mass spectrometry [18]. Recently, a comparative study between predictive capacities of SDHB immunohistochemistry and SFR methods has been reported [25]. In a general set of PGLs/PCCs, metabolite measurements showed higher specificity than SDHB immunohistochemical staining (99.2% versus 92.5%), but their sensitivity was comparable (88.1% versus 85.2%). However, the sensitivity of both methods was lower for HNPGLs than for other PGLs and PCCs. Thus, HNPGLs appear to be more difficult to diagnose using IHC and SFR methods, and need detailed study.

In this work, we first performed a correlation analysis between variants in the *SDHx* genes and their expression at the protein level in a representative set of CPGLs. The mutation status of the *SDHx* genes was determined using exome sequencing, and protein levels were estimated with IHC. We also evaluated the possibility of SDHA/SDHB immunostaining use to predict variants in any of the *SDHx* genes in CPGLs. We have found that SDHB staining does not always correlate with *SDHx* variants, while several studies proposed the immunohistochemistry of the SDHB subunit as a useful instrument for the prediction of *SDHx* mutation status. These important results indicate the necessity of genetic testing of *SDHx* variants along with IHC study in CPGLs.

## 2. Results

### 2.1. Pathogenic/Likely Pathogenic Variants of Susceptibility Genes in CPGLs

**Figure 1:**
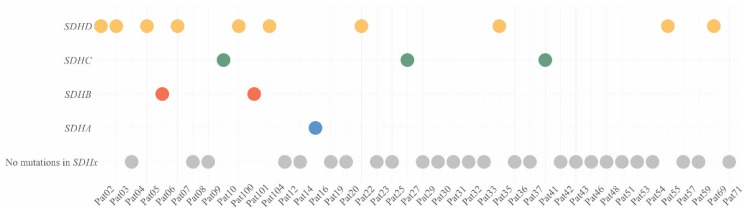
Distribution of *SDHx* variants in patients with CPGLs. Yellow dots—patients with *SDHD* variants, green dots—patients with *SDHC* variants, red dots—patients with *SDHB* variants, blue dot—patient with *SDHA* variant, grey dots—patients with no mutations in *SDHx*.

We used open-sources (ClinVar [26], dbSNP [27], and COSMIC [28]), prediction algorithms (SIFT [29], PolyPhen2 [30], MutationTaster [31], and LRT [32]), as well as position region conservation score (PhastCons [33] and PhyloP [34]) for interpretation of the variants, and analysis was only performed for variants with less than 1% population frequency. Pathogenicity of new variants was assessed using the criteria of the American College of Medical Genetics and Genomics and the Association for Molecular Pathology (ACMG-AMP) [35]. Pathogenic/likely pathogenic variants in the *SDHx* genes were found in 16 out of 42 (38%) patients with CPGLs (Table 1, Figure 1). Among the 42 studied patients, ten had variants in the *SDHD* gene (24%), two were characterized with variants in *SDHB* (5%), three had variants in *SDHC* (7%), and one carried a variant in *SDHA* (2%).

In addition, we have analyzed the mutation status of *RET*, *VHL*, *TMEM127*, *MAX*, *IDH1*, *IDH2*, *FH*, and *SLC25A11*, as well as of genes belonging to the family of succinate dehydrogenase complex assembly factors (*SDHAF1*, *SDHAF2*, *SDHAF3*, and *SDHAF4*), which are the main susceptibility genes for CPGLs (Table 1). Pathogenic/likely pathogenic variants were found in *RET* and *IDH1*; no variants in *VHL*, *TMEM127*, *MAX, IDH2*, *FH*, *SLC25A11,* and *SDHAF1*-*4* genes were identified.

Pathogenic variants in *RET* were observed in three (7%) patients with CPGLs. A pathogenic variant in *RET* NM_020975: c.2372A > T, p.(Tyr791Phe) (chr10: 43613908, rs77724903;13936) was revealed in two patients who also carried pathogenic/likely pathogenic variants in *SDHA* (Pat16) and *SDHD* (Pat35). In Pat27, we found a pathogenic variant in *RET* NM_020975: c.2944C > T, p.(Arg982Cys) (chr10: 43620335, rs17158558) that was corepresented with a likely pathogenic frameshift variant in the *SDHC* gene.

A likely pathogenic variant in *IDH1* NM_005896: c.394C > T, p.(Arg132Cys) (chr2: 209113113, rs121913499) has been observed in one (2%) patient (Pat31) together with no mutations in the *SDHx* genes.

### 2.2. Correlation of SDHx Mutation Status with Their Immunostaining

**Figure 2:**
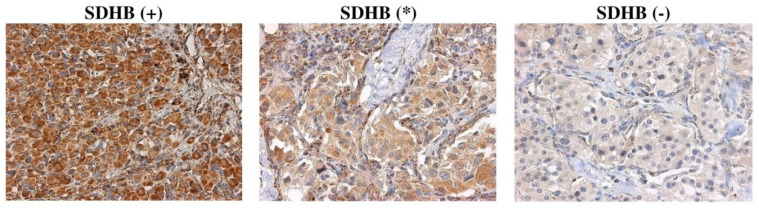
Representative images of SDHB immunostaining in CPGLs. (+) positive, (*) weak diffuse, (-) negative. Magnification x400.

Immunohistochemical staining was performed for SDHA, SDHB, SDHC, and SDHD subunits on 42 CPGL samples (Supplementary File S1). SDHB staining was assessed as follows: (+) Positive as granular cytoplasmic staining of tumor cells in parallel with the same intensity staining of internal positive control (endothelial cells); (-) negative as completely absent cytoplasmic staining together with staining of internal positive control; (*) weak diffuse as a cytoplasmic blush lacking definite granularity contrasting the strong granular staining of internal positive control (Figure 2). Immunostaining of SDHA, SDHC, and SDHD was scored as positive or negative in the same manner as SDHB.

In the majority of *SDHx*-mutated tumors (94%, 15/16), we detected negative or weak diffuse staining of SDHB (Supplementary Table S1). The Spearman’s rank correlation coefficient (*r_s_*) between the presence of mutations in any *SDHx* genes and negative or weak diffuse SDHB staining was 0.51, *p* ≤ 0.05.

Negative or weak diffuse SDHB staining was found in nine out of ten cases with pathogenic/likely pathogenic *SDHD* variants; one *SDHD*-mutated tumor showed positive staining of all SDH subunits. In all the samples, SDHD was positively stained.

In two out of three samples with pathogenic/likely pathogenic variants in the *SDHC* gene, we identified weak diffuse SDHB staining and simultaneous positive SDHC expression. In one case, negative immunohistochemical staining for both SDHB and SDHC was found.

All samples with pathogenic/likely pathogenic variants in the *SDHB* gene showed negative SDHB staining.

A pathogenic/likely pathogenic variant in the *SDHA* gene was identified only in one patient. We observed both weak diffuse SDHB staining and negative SDHA expression in this sample.

Among twenty-six CPGLs with no pathogenic/likely pathogenic variants in the *SDHx* genes, negative or weak diffuse SDHB staining was observed in eleven cases (42%). Fifteen samples were immunopositive for all SDH subunits.

Three tumors with pathogenic variants of the *RET* gene, which were corepresented with *SDHA*, *SDHC*, and *SDHD* variants, have been characterized by negative or weak diffuse SDHB staining. Positive immunoreactivity was found in one patient with a likely pathogenic variant in the *IDH1* gene occurring with no variant in *SDHx*.

### 2.3. Calculation of Diagnostic Accuracy

To determine the ability of SDHB immunohistochemistry discriminating *SDHx*-mutation carriers, we measured the sensitivity, specificity, and diagnostic effectiveness (accuracy) according to the following formulas:

Sensitivity  =  TP/TP  +  FN,

Specificity  =  TN/TN  +  FP,

Accuracy  =  TN  +  TP/TN  +  TP  +  FN  +  FP,

where TP (true positives)—positively diagnosed subjects with the disease, FN (false negatives)—negatively diagnosed subjects with the disease, TN (true negatives)—negatively diagnosed subjects without the disease, and FP (false positives)—positively diagnosed subjects without the disease.

In CPGLs, SDHB immunohistochemistry showed a sensitivity of 94% (15/16) and specificity of 58% (15/26). The diagnostic effectiveness of this method was 71% (30/42).

## 3. Discussion

Tumor cells are well-known to have alterations in energy metabolism that are exemplified by the Warburg effect [36,37]. A metabolic shift from mitochondrial respiration to glycolysis can be caused by mitochondrial dysfunction or by the reduction in its activity [38,39]. SDH has a critical role in mitochondrial metabolism; disruption of the SDH complex leads to abnormal accumulation of succinate in the cytosol, reprogramming of the energy metabolism, increased ROS production, stabilization of hypoxia-inducible factors (HIFs), and altered gene expression (in particular, for HIF targets) [40]. All of these changes can trigger neoplastic growth [38,41]. SDH abnormalities are associated with a tumorigenesis risk, including the development of PGLs/PCCs, renal and thyroid cancer, as well as composite PGLs/gastrointestinal stromal tumors (GISTs)/pulmonary chondromas (Carney triad) and PGLs/GISTs (Carney–Stratakis syndrome) [42].

*SDHx* are the most commonly mutated genes in PGLs/PCCs [43]. Variants in *SDHD* are more frequently observed in HNPGLs, followed by *SDHB* and *SDHC* mutations [44,45]. *SDHA* variants show extremely low penetrance in HNPGLs [10]. We obtained similar results; however, *SDHC* variants were found more often than *SDHB* variants. Previously, it has been reported that *SDHC* mutations are mainly associated with the development of CPGLs, explaining this difference [46].

Mutations in any of the *SDHx* genes can cause a destabilization of the SDH complex, loss of its enzymatic activity, and a disruption in the electron transport function [47,48,49,50]. Numerous studies have reported a changed expression pattern of SDHB presented as negative or weak diffuse immunostaining in tumors with *SDHA*-, *SDHB*-, *SDHC*-, and *SDHD* mutations [24,51,52,53]. It was shown that negative SDHB staining is more commonly associated with mutations in *SDHB*, whereas weak diffuse staining often occurs in *SDHD*-mutated tumors [52]. We also detected loss of SDHB expression in all patients with *SDHB* variants and weak diffuse SDHB staining in the majority of *SDHD* mutation carriers that support this finding. Studied patients carrying variants in *SDHC* showed both negative and weak diffuse SDHB staining. Notably, a number of authors interpreted SDHB staining only as positive or negative and considered a weak diffuse expression pattern as negative. Generally, both patterns indicate SDH deficiency, which is a surrogate marker for *SDHx* germline mutations almost always causing the gene biallelic inactivation [23]. Somatic events leading to biallelic inactivation have been rarely reported for the *SDHx* genes [23]. In the study, we used an archival collection of CPGLs for which paired normal tissues were unavailable; therefore, germline and somatic mutation status could not be estimated. However, based on this conception, we can suppose that in the majority of studied patients with SDH deficiency, the mutations of *SDHx* genes are germline. In one patient with a novel likely pathogenic frameshift *SDHD* variant, we found retention of SDHB expression. Possibly, this variant does not have a high impact on the protein structure or it occurs in one allele of the gene.

In a patient with a pathogenic *SDHA* variant, we have seen completely absent SDHA expression and weak diffuse SDHB immunostaining that is in accordance with the literature. Direct correlation with the presence of the gene mutation and loss of the protein expression is observed only for *SDHA*. Negative SDHA expression is defined both when mutation leads to the truncated protein and owing to missense mutation [12,54]. SDHB expression at the same time also becomes negative in SDHA-mutated tumors, supported by almost all reported cases (including our results) [12,24,54,55]. Moreover, *SDHA* mutation is a rare event in PGLs; therefore, the use of SDHB immunohistochemistry seems to be more expedient than SDHA/SDHB immunohistochemistry for prediction of mutations in any *SDHx* genes.

The loss of SDHC expression was revealed in one out of three patients with variants in the *SDHC* gene. This variant, NM_003001.3: c.224G > A, p.(Gly75Asp) (chr1: 161310428б rs786205147;189841), was described in the ClinVar database as a germline likely pathogenic variant associated with the hereditary cancer-predisposing syndrome and Carney triad with no experimental evidence of its pathogenicity to date. In this patient, negative SDHB staining was also determined. Therefore, we can suggest that, except for SDHA, no evident correlations have been found between negative SDHC and SDHD immunohistochemistry and the presence of pathogenic variants in the corresponding genes.

Among 42 patients with CPGLs, we revealed pathogenic *RET* variants in three cases and a likely pathogenic *IDH1* variant in one patient. *RET* variants were presented in *SDHx*-mutated tumors that showed negative or weak diffuse SDHB staining. *SDHx*-mutations seem to be the main drivers of SDH efficiency; therefore, we cannot correctly assess the correlation of identified *RET* variants with the SDHB immunohistochemistry in these samples. The presence of the *IDH1* variant was not associated with the changed immunostaining of any SDH subunits. However, more cases are needed to assess the impact of *IDHx* mutations on the stability of the SDH complex.

Despite the great results showing a high correlation of SDHB immunohistochemistry with the presence of *SDHx* variants (94%), negative or weak diffuse SDHB staining has also been found in 42% of tumors without pathogenic/likely pathogenic variants in any *SDHx* genes. In this case, SDH deficiency can be caused by mutations in the DNA regions, which have not been screened, or epimutations.

Given these data, we presumed that SDHB immunohistochemistry could be used for the initial assessment of *SDHx* variants in CPGLs with genetic testing in parallel. Additional SDHA staining increases the cost of the IHC analysis, but among PGLs/PCCs, SDHA mutation frequency is extremely low, and in the majority of such cases, negative or weak diffuse SDHB staining is also observed.

## 4. Materials and Methods

### 4.1. Tumor Samples and Patients

A total of 42 carotid paraganglioma samples (archive material) were used in this study. The formalin-fixed paraffin-embedded (FFPE) tumor tissues were collected in the Vishnevsky Institute of Surgery, Ministry of Health of the Russian Federation. Tumors were obtained from patients who did not receive radiotherapy or chemotherapy before surgery. Samples have no less than 80% of tumor cells. All the patients provided written informed consent. The study was approved by the ethics committee from the Vishnevsky Institute of Surgery with ethics committee approval no. 007/18, 02.10.2018 and performed according to the Declaration of Helsinki (1964). The clinicopathologic characteristics of the patients with CPGLs are presented in Table 2.

### 4.2. DNA Extraction

DNA was extracted from tumor tissues using a High Pure FFPET DNA Isolation Kit (Roche, Basel, Switzerland) according to the manufacturer’s instructions. The quantification of isolated DNA was performed with a Qubit 2.0 Fluorometer (Thermo Fisher Scientific, Waltham, MA, USA). DNA quality was assessed by quantitative PCR (qPCR) using QuantumDNA Kit (Evrogen, Moscow, Russia).

### 4.3. Exome Sequencing

Exome libraries were prepared from DNA using a Rapid Capture Exome Kit (Illumina, San Diego, CA, USA) or TruSeq Exome Library Prep Kit (Illumina), according to the guidelines. Capture probes covered the same DNA regions in both kits (predominantly gene-coding regions). Library quantification was carried out using both Qubit 2.0 Fluorometer and qPCR. A quality assay of the libraries was performed on an Agilent 2100 Bioanalyzer (Agilent Technologies, Santa Clara, CA, USA). High-throughput sequencing of the libraries was performed on a NextSeq 500 System (Illumina) in a paired-end mode of 76 × 2 bp. The average coverage for each sample was at least 300×. In this study, we used exome data of CPGL samples that were previously sequenced; raw sequence reads for Pat02–Pat51 are available at NCBI Sequence Read Archive (SRA) BioProject PRJNA411769 [56], and sequence data for Pat100–Pat104 are available at SRA BioProject PRJNA476932 [57]. Raw sequence data from an expanded set of CPGL samples (Pat53–Pat71) were added to the NCBI SRA BioProject PRJNA411769.

Bioinformatic analysis of exome sequencing data was carried out in the R environment. Raw reads were qualified using FASTQC (v. 0.11.9, Babraham Bioinformatics, Cambridge, UK). Quality trimming (less than Q20) and adapter removal were done with Trimmomatic (v. 0.39, USADEL LAB, Jülich, Germany) [58]. Then, reads were mapped to the reference human genome GRCh37.75/hg19 using BWA (v. 0.7.17, Wellcome Trust Genome Campus, Cambridge, UK) [59]. SAMtools (v. 1.10, Wellcome Trust Genome Campus, Cambridge, UK) [60] was used for BAM file sorting, and files were then processed with picard-tools (v. 2.23.4, Broad Institute, Cambridge, MA, USA). Base quality score recalibration was done with GATK4 (v. 4.1.2, Broad Institute, Cambridge, MA, USA) [61] and dbSNP (common variants 2015-06-05, Bethesda, MD, USA). Variant detection was carried out with GATK HaplotypeCaller [61]. We used GATK StrandBiasBySample, StrandOddsRatio, and BaseQualityRankSumTest to exclude false positives. Additionally, we excluded mis-sequenced single-nucleotide variants (SNVs) in polyN motifs, such as GGGTG >GGGGG, CCCCG >CCCCC, and others. Variants were annotated with Annovar (v. 20200316, Center for Applied Genomics, Philadelphia, PA, USA) [62]. VCF files included data on allele frequency (1000 Genomes Project [63], ExAC [64], gnomAD [65], Kaviar [66], and ESP-6500 [http://evs.gs.washington.edu/EVS/]), variant annotation (ClinVar, dbSNP, and COSMIC), position region conservation score (PhastCons and PhyloP [both PHAST v. 1.5, Siepel Lab, Cold Spring Harbor, NY, USA]), localization of variants in protein domains (InterPro [v. 81.0, Wellcome Genome Campus, Hinxton, Cambridgeshire, UK] [67]), as well as on pathogenicity prediction (SIFT [v. 6.2.1, Fred Hutchinson Cancer Research Center, Seattle, WA, USA], PolyPhen2 [v. 2.2.2, Harvard Medical School, Boston, MA, USA], MutationTaster [v. 2013-03-20, “Charité–Universitätsmedizin Berlin”, Berlin, Germany], and LRT [v. 0.2, Tel-Aviv University, Tel Aviv, Israel]).

### 4.4. Immunohistochemistry

IHC staining was used to analyze *SDHx* gene expression at the protein level. Sections (3–5 µm) from FFPE samples were prepared on glass slides using an HM 355S Automatic Microtome (Thermo Fisher Scientific) and then stained with hematoxylin-eosin (H&E) for histomorphological analysis. Deparaffinization of the sections was performed with xylene, with further rehydration in decreasing alcohol concentrations (absolute, 90%, 70%, and 50%) and washing in distilled water. Immunoreactions were performed in a serial manner using primary antibodies for all four SDH subunits (SDHA, monoclonal, clone 2E3GC12FB2AE2; SDHB, monoclonal, clone 21A11AE7; SDHC, monoclonal, clone EPR11035(B); SDHD, polyclonal) from Abcam (Cambridge, United Kingdom) on a Lab Vision Autostainer 360-2D (Thermo Fisher Scientific), according to the manufacturer’s instructions. Reactions continued in a ready-to-use visualization system Histofine DAB-2V (Nichirei Biosciences, Tokio, Japan) with universal chromogen-labeled (3,3′-diaminobenzidine, DAB) secondary antibodies. Additional Mayer’s hematoxylin staining was performed. Samples incubated without primary antibodies were used as the negative controls (Supplementary Figure S1). Granular cytoplasmic staining of SDH subunits in endothelial cells was used as a positive internal control. The slides were visualized using an Axio Imager 2 (Carl Zeiss Microscopy, Jena, Germany).

### 4.5. Correlation Analysis

Correlation analysis between SDHB staining and the presence of mutations in any *SDHx* genes was performed using the Spearman’s rank correlation test with STATISTICA 10 (StatSoft Inc., Tulsa, OK, USA).

## 5. Conclusions

This is the first study on the correlation between *SDHx* mutation status and their protein expression, respectively estimated with exome sequencing and IHC in a representative set of CPGLs. It has previously been reported that negative or weak diffuse SDHB staining has high sensitivity and specificity for the prediction of mutations of *SDHx* in PGLs/PCCs. However, our study showed that altered SDHB immunostaining widely occurs in tumors that do not carry pathogenic/likely pathogenic variants in the genes. These divergent results could be explained by the fact that earlier studies focused on PGLs/PCCs or all HNPGLs, but not only on CPGLs. Nevertheless, the sensitivity of the method remains high. Based on the collected data, we believe that SDHB immunohistochemistry could be used for primary identifications of patients potentially carrying *SDHx* variants who should be further referred for genetic testing.

## Figures and Tables

**Table 1:** Pathogenic/likely pathogenic variants in *SDHx*, *RET*, and *IDH1* genes found in patients with carotid paragangliomas (CPGLs).

Pat	Gene	dbSNP ID	GeneBank	Pos	NC Change	AA Change	ClinSig	
Pat16	*SDHA*	rs1061517;239661	NM_004168	chr5: 218471	c.1A > G	p.Met1?	P/LP	
Pat06	*SDHB*	rs727503415;165180	NM_003000	chr1: 17359564	c.277T > C	p.Cys93Arg	P	
Pat101		rs74315370;142763		chr1: 17371320	c.136C > T	p.Arg46*	P	
Pat10	*SDHC*	-	NM_003001	chr1: 161310387	c.183G > A	p.Trp61*	LP*	
Pat27		-		chr1: 161332121	c.409delT	p.Trp137fs	LP*	
Pat41		rs786205147;189841		chr1: 161310428	c.224G > A	p.Gly75Asp	LP	
Pat05 Pat22 Pat100 Pat104	*SDHD*	rs104894302	NM_003002	chr11: 111959726	c.305A > G	p.His102Arg	LP	
Pat02		rs80338843;6893		chr11: 111958640	c.112C > T	p.Arg38*	P	
Pat03		-		chr11: 111957643	c.13dupT	p.Trp5fs	LP*	
Pat07		rs104894307;6911		chr11: 111957632	c.1A > G	p.Met1?	P	
Pat35		-		chr11: 111959626	c.205G > T	p.Glu69*	P*	
Pat55		-		chr11: 111965547	c.335_338del	p.Thr112fs	LP*	
Pat69		-		chr11: 111959637	c.217dupA	p.Ser33fs	LP*	
Pat31	*IDH1*	rs121913499	NM_005896	chr2: 209113113	c.394C > T	p.Arg132 Cys	LP	
Pat16 Pat35	*RET*	rs77724903;13936	NM_020975	chr10: 43613908	c.2372A > T	p.Tyr791Phe	P	
Pat27		rs17158558		chr10: 43620335	c.2944C > T	p.Arg982Cys	P	

Pat—patient; Pos—position; NC—nucleotide; AA—amino acid; ClinSig—clinical significance; P—pathogenic; LP—likely pathogenic. Clinical significances of the variants were interpreted using the ClinVar database and (*) ACMG-AMP guideline.

**Table 2:** Clinicopathologic characteristics of patients with CPGLs.

Characteristic	Number of patients, n
**Total number**	42
**Age, yr**	
<40	15
≥40	27
**Sex**	
Female	28
Male	14
**Multifocal tumor**	2
**Bilateral CPGL**	1
**Recurrent patients**	1
**Familial history**	
Positive	1
Negative	0
N/A	41

## Supplementary Materials

Can be found at https://www.mdpi.com/1422-0067/21/18/6950/s1.

## Abbreviations

CPGL	carotid paraganglioma
*SDHx*	genes encoding for succinate dehydrogenase subunits
SDH	succinate dehydrogenase
IHC	immunohistochemistry
SFR	succinate to fumarate ratio
