# Supplementary material for: Immunohistochemistry and Mutation Analysis of SDHx Genes in Carotid Paragangliomas
Source: Int J Mol Sci. 2020 Sep 22;21(18):6950. doi: 10.3390/ijms21186950 (PMC7576476; doi:10.3390/ijms21186950)

Pat02

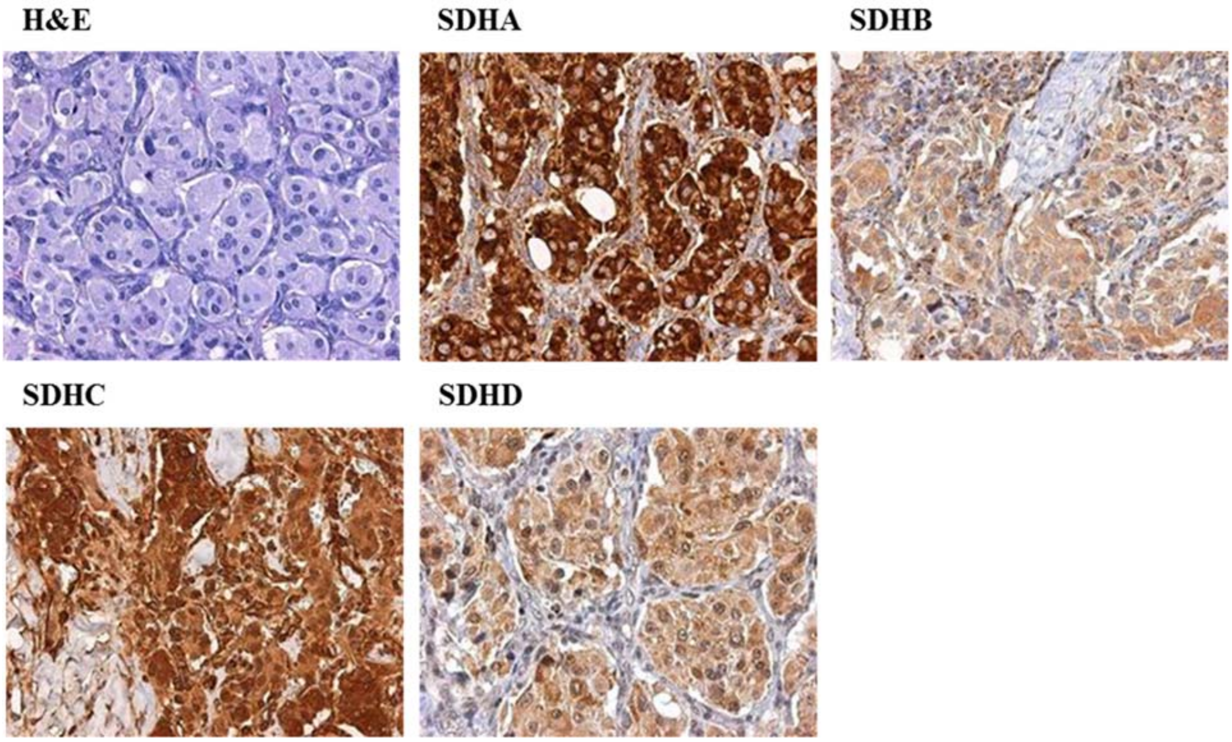

Pat03

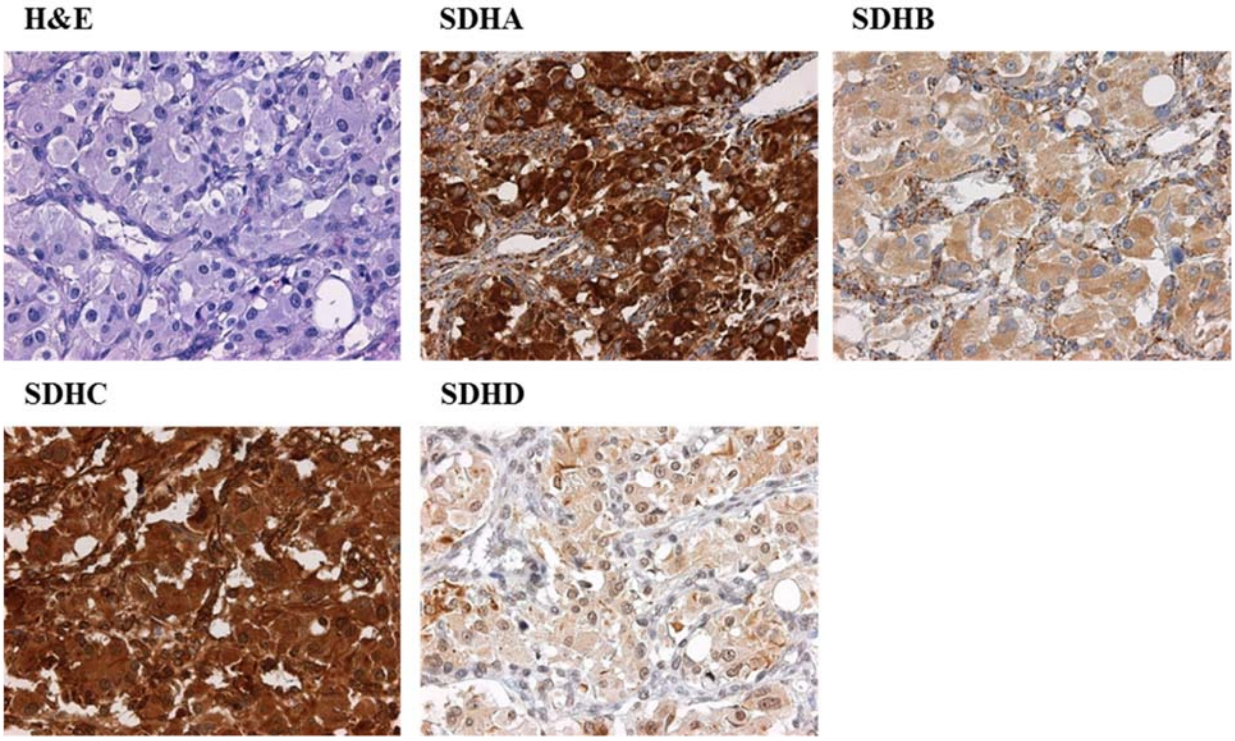

Pat04

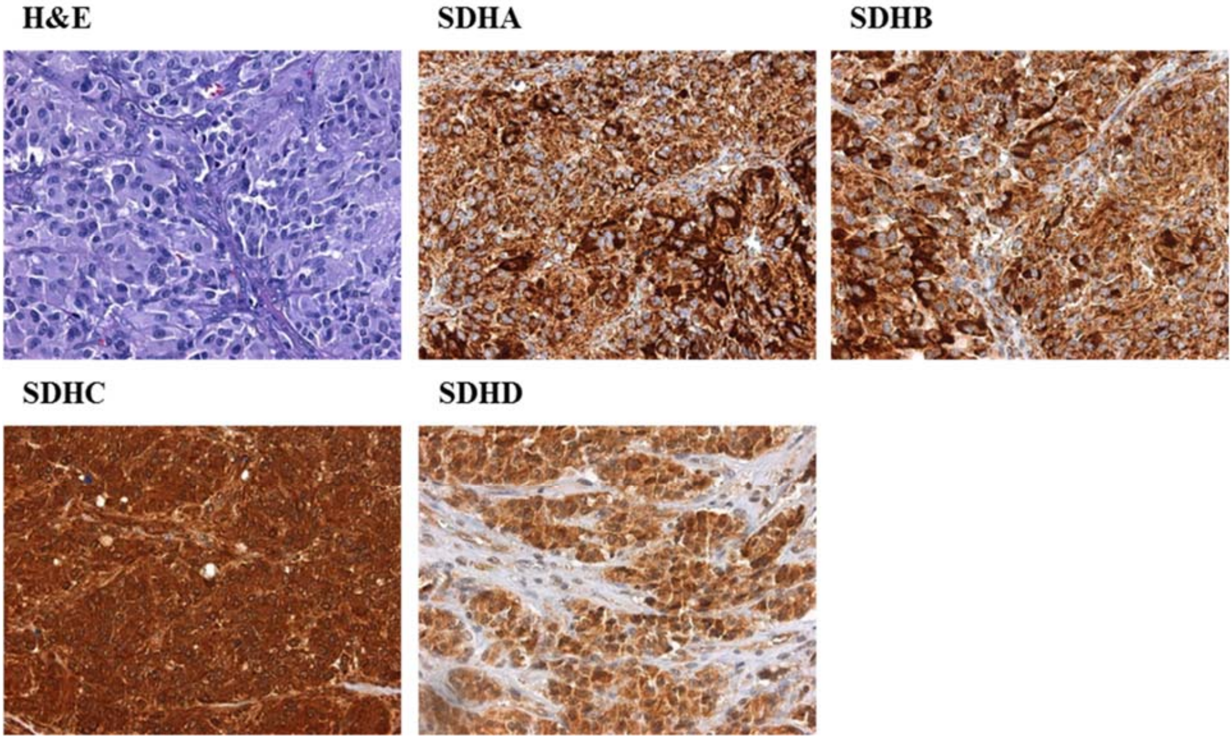

Pat05

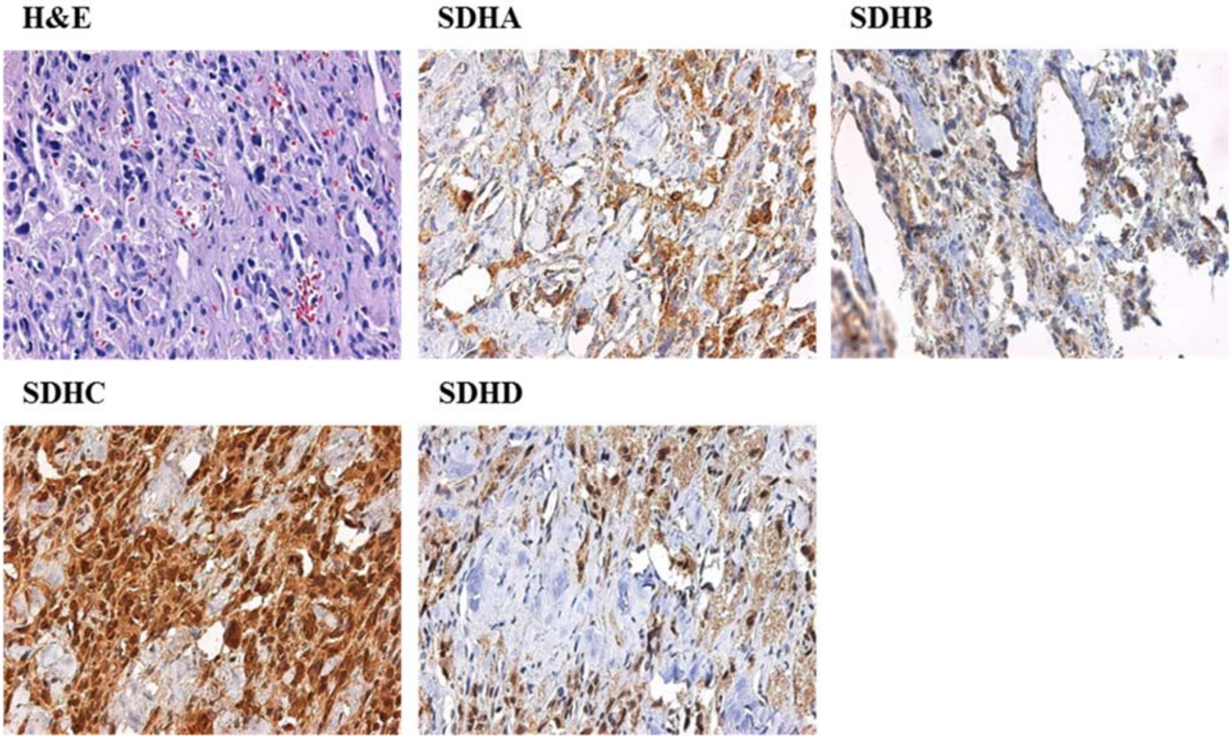

Pat06

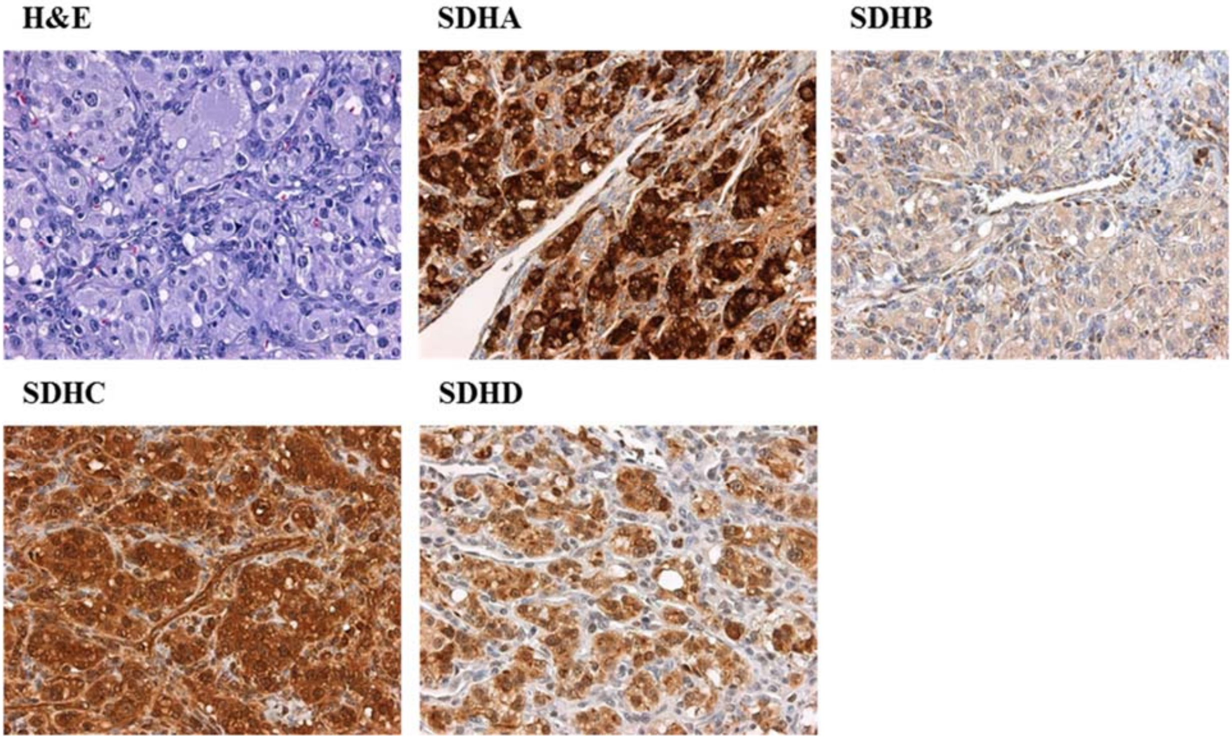

Pat07

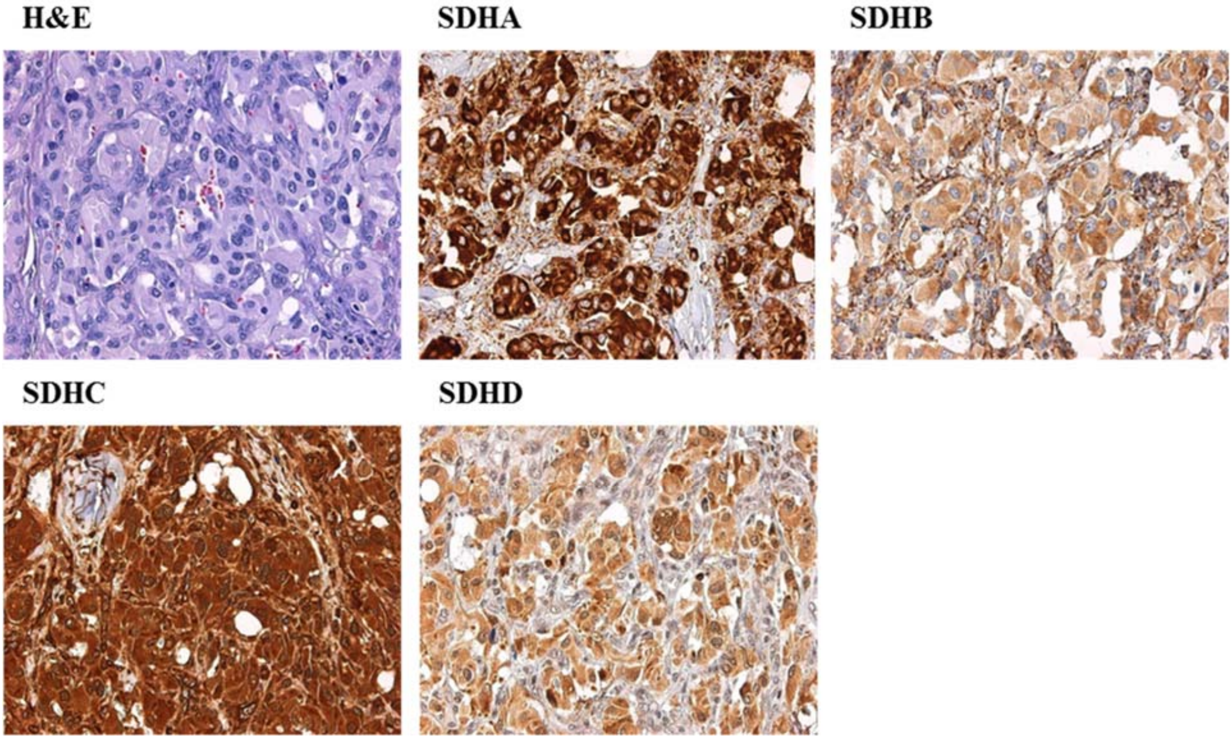

Pat08

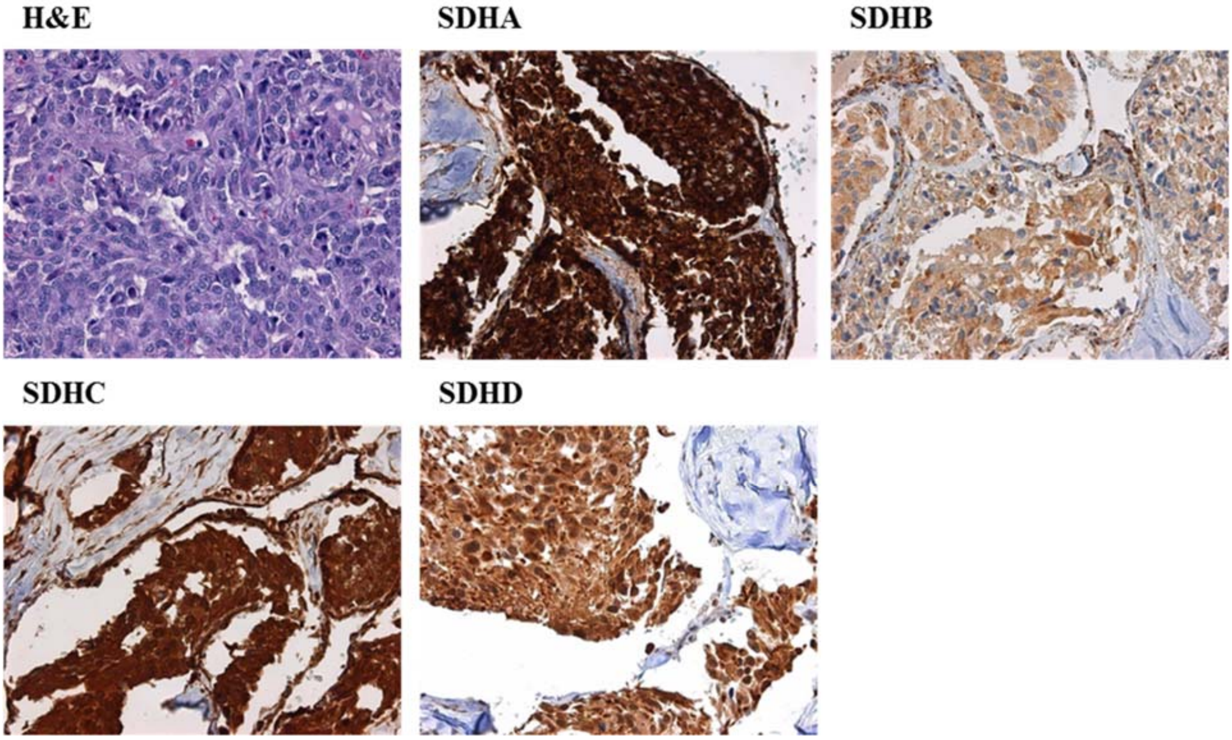

Pat09

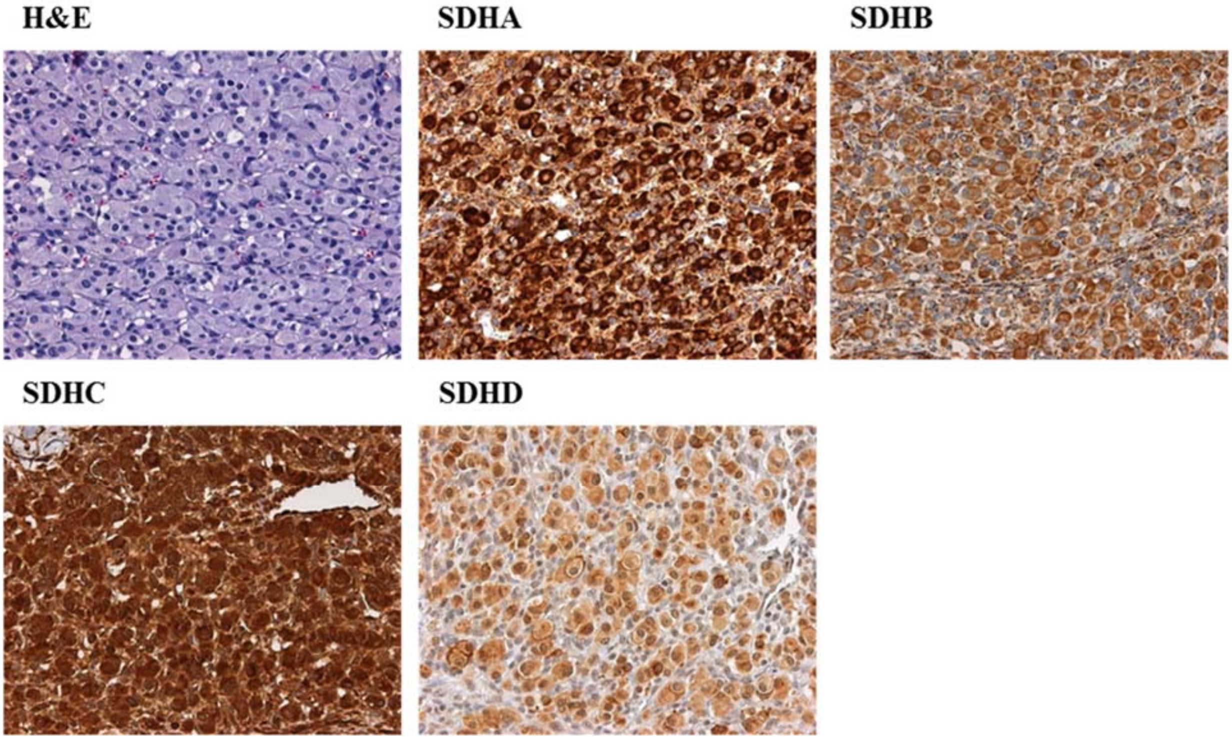

Pat10

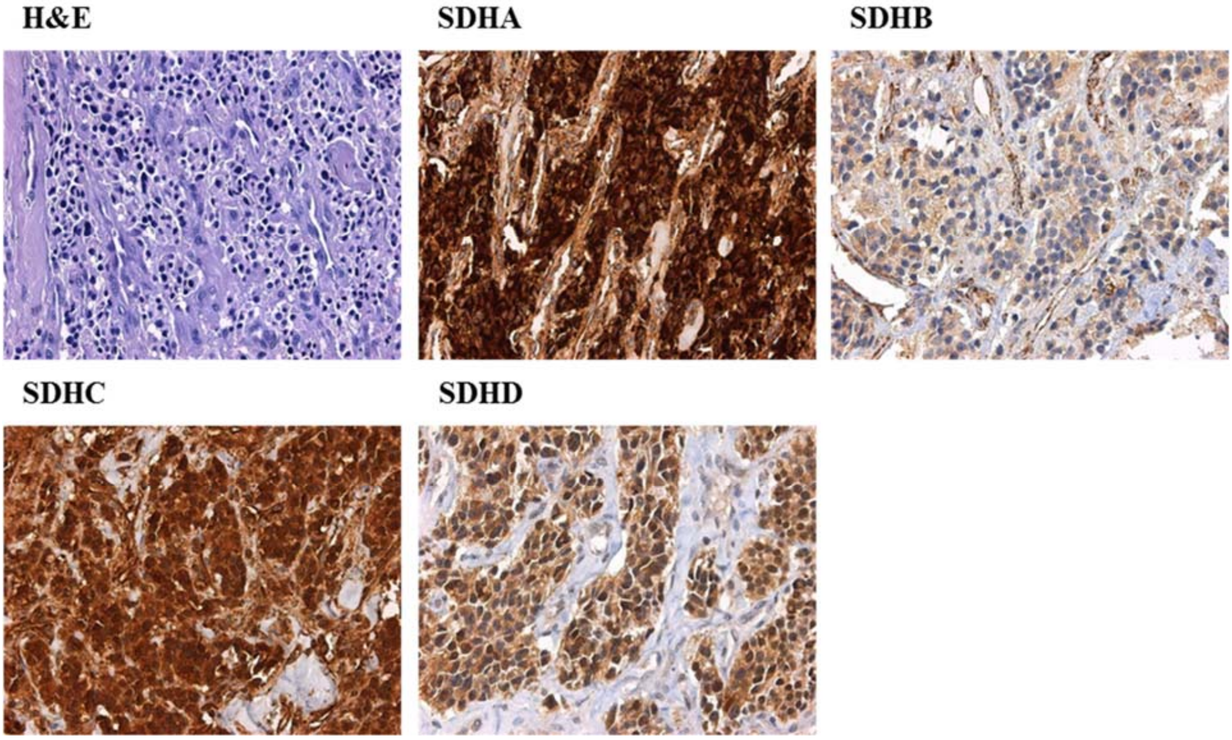

Pat12

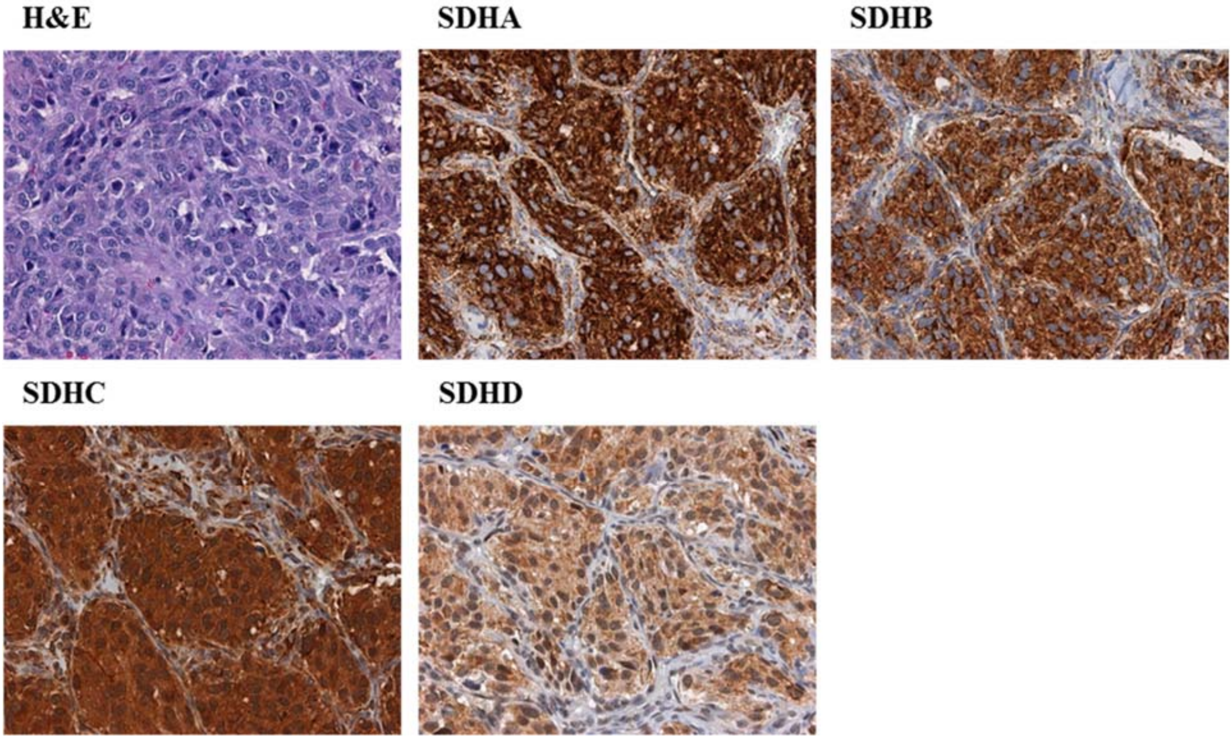

Pat14

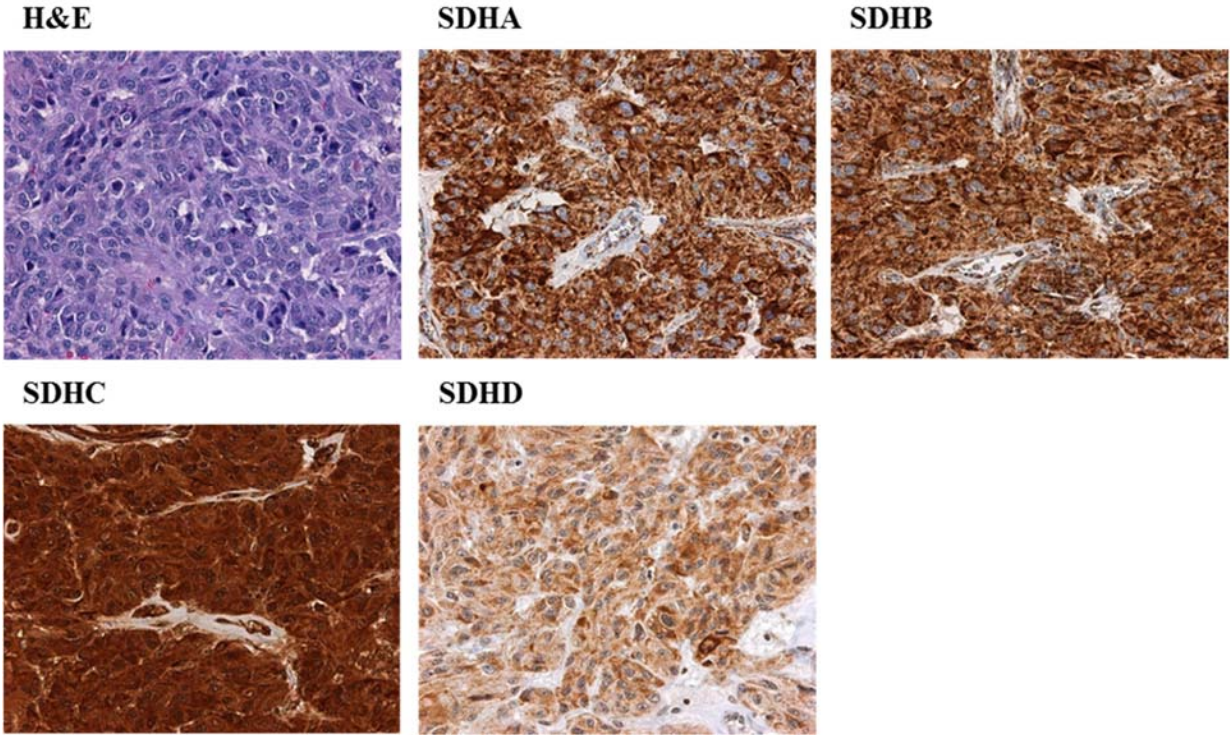

Pat16

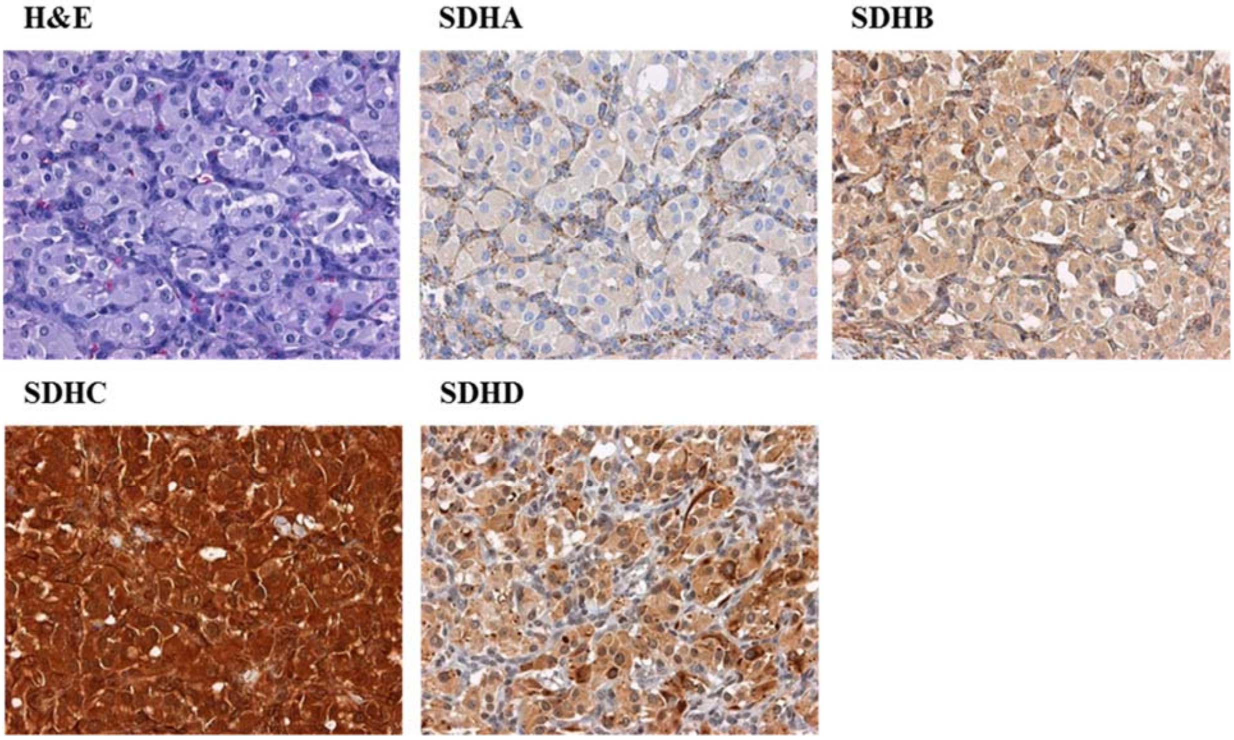

Pat19

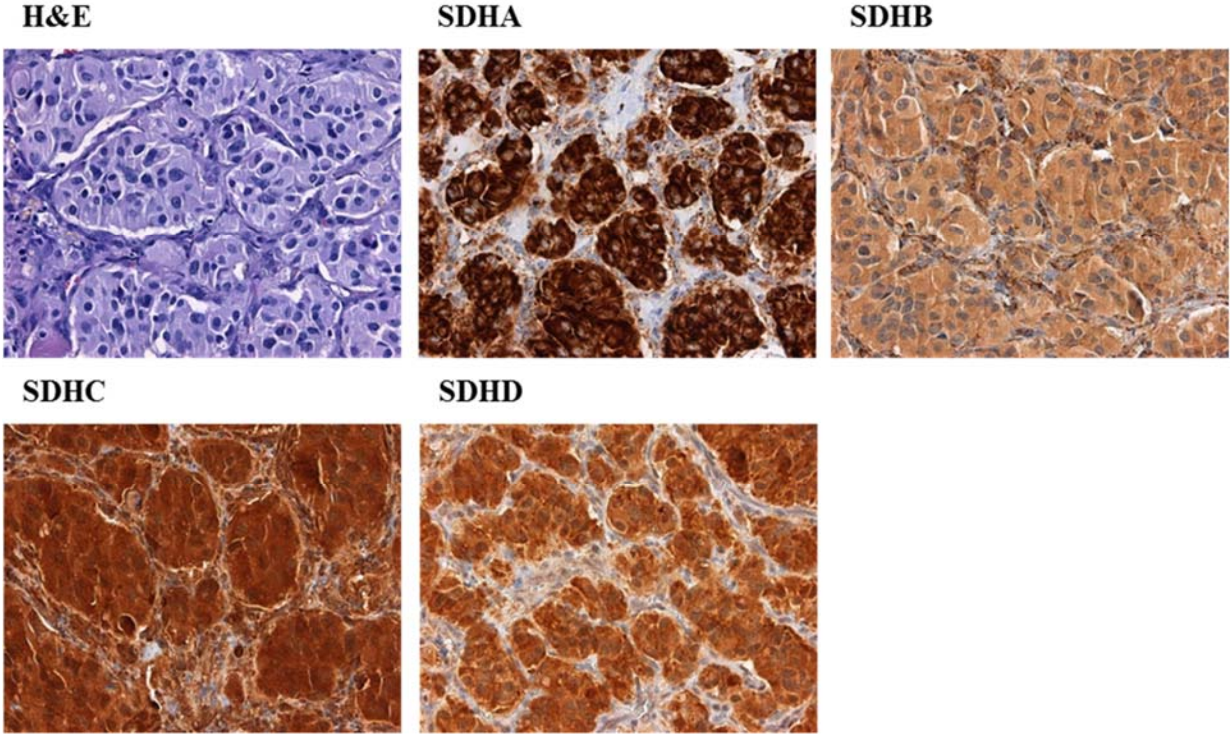

Pat20

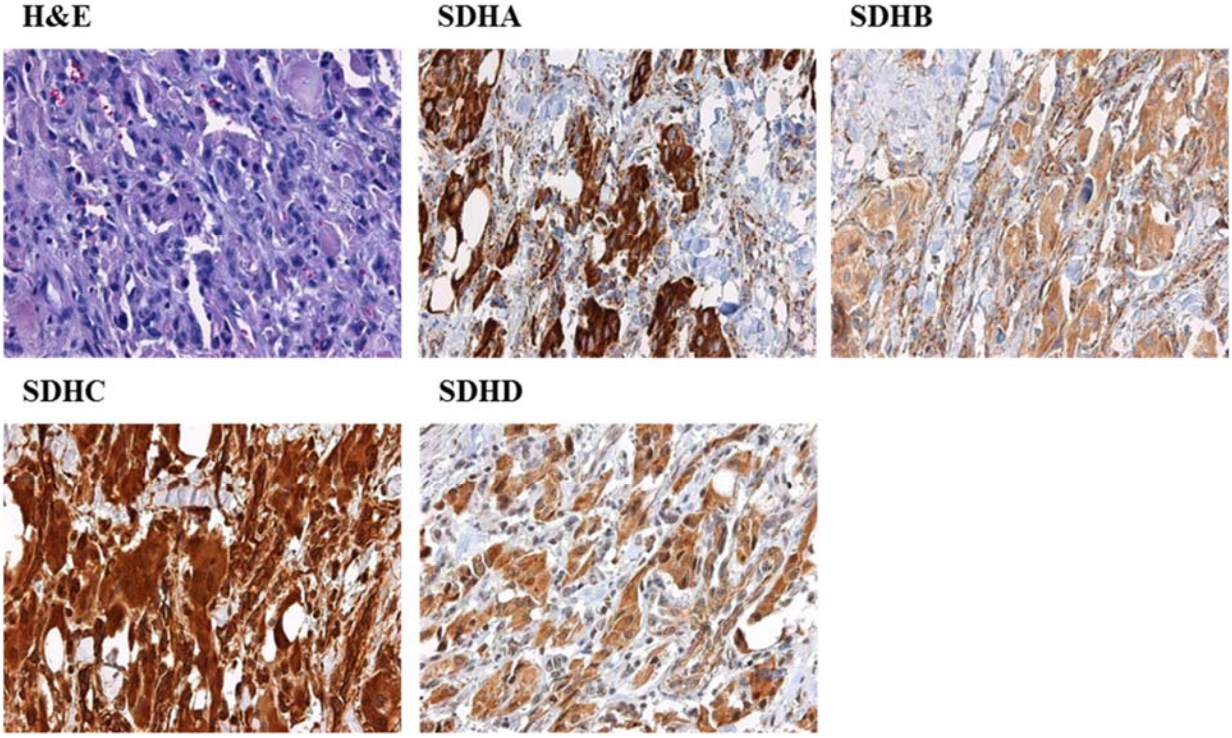

Pat22

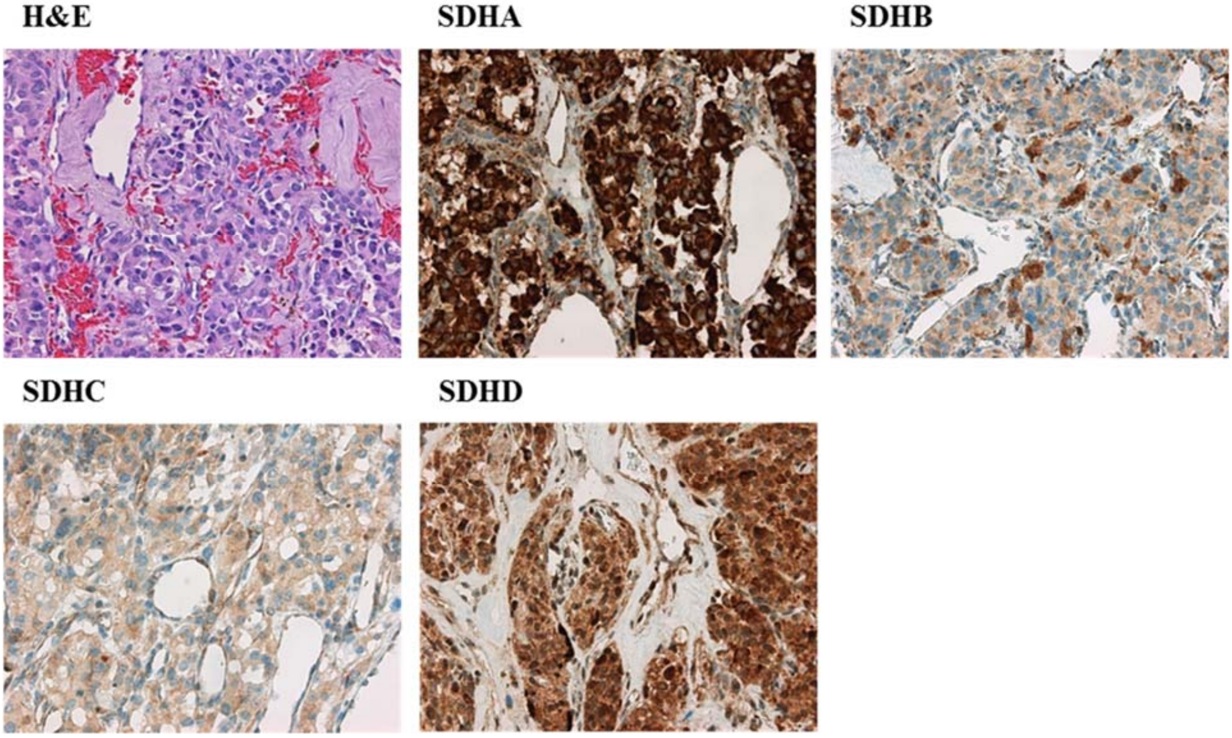

Pat23

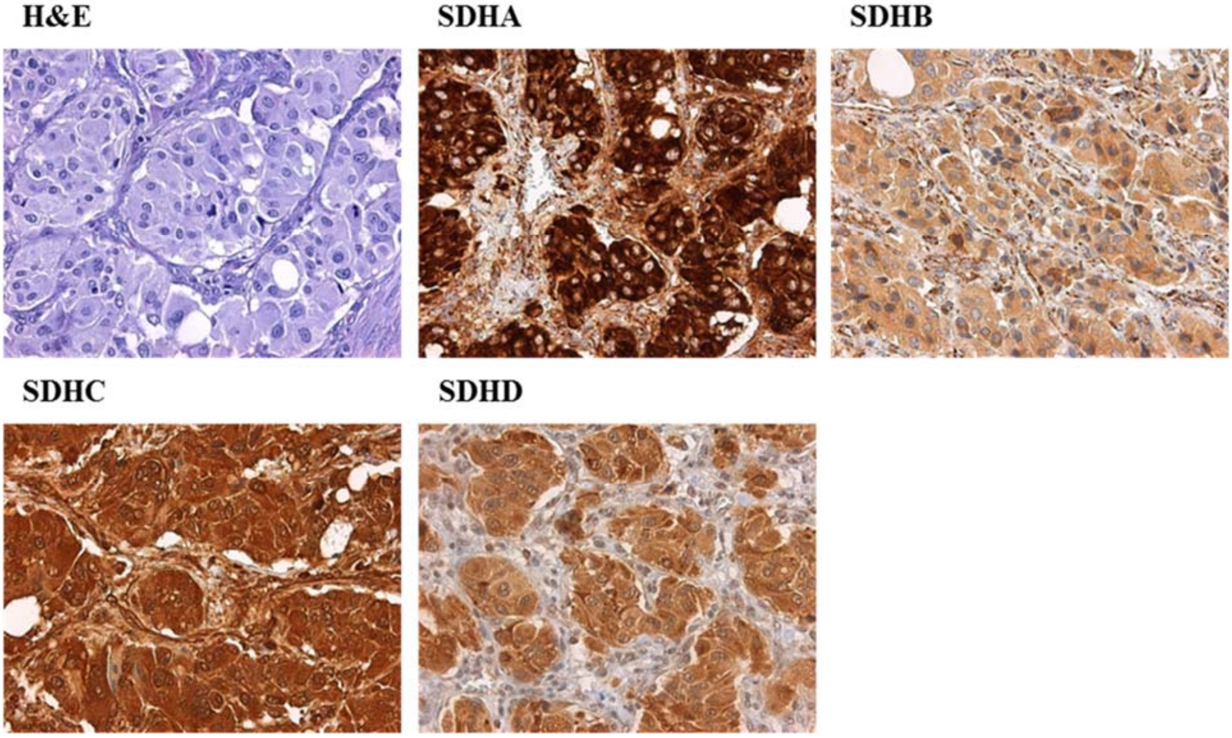

Pat25

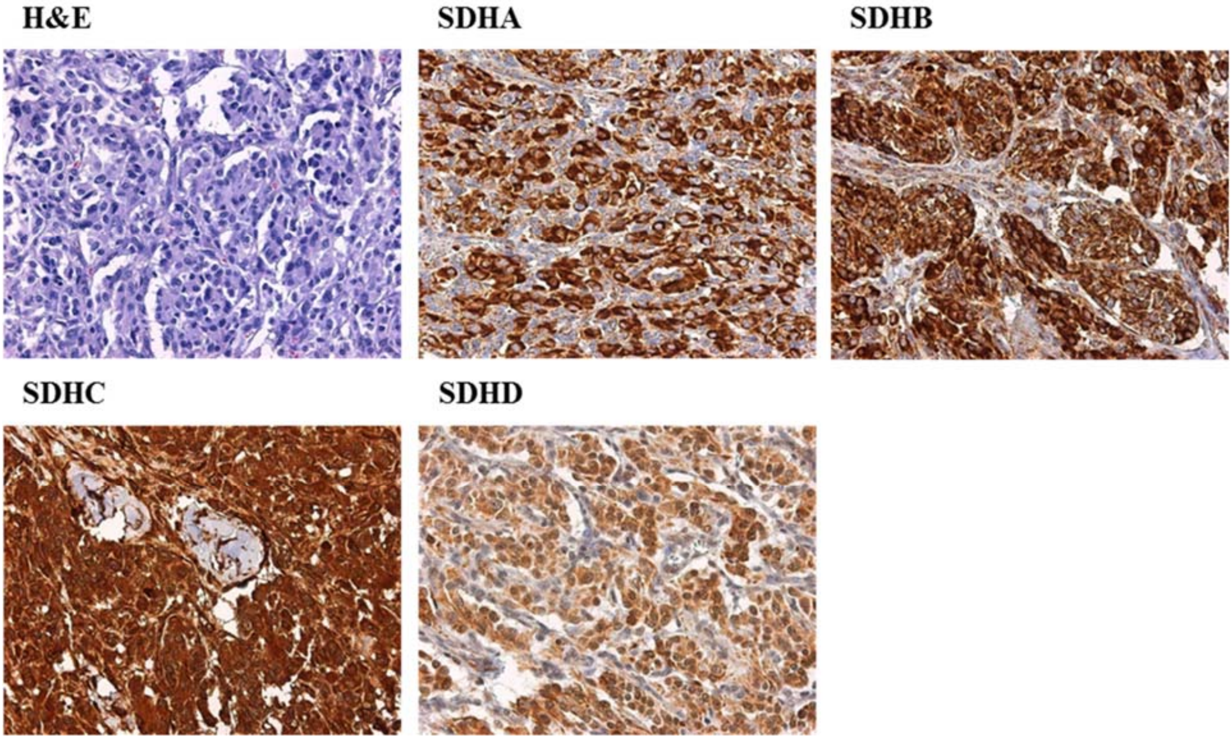

Pat27

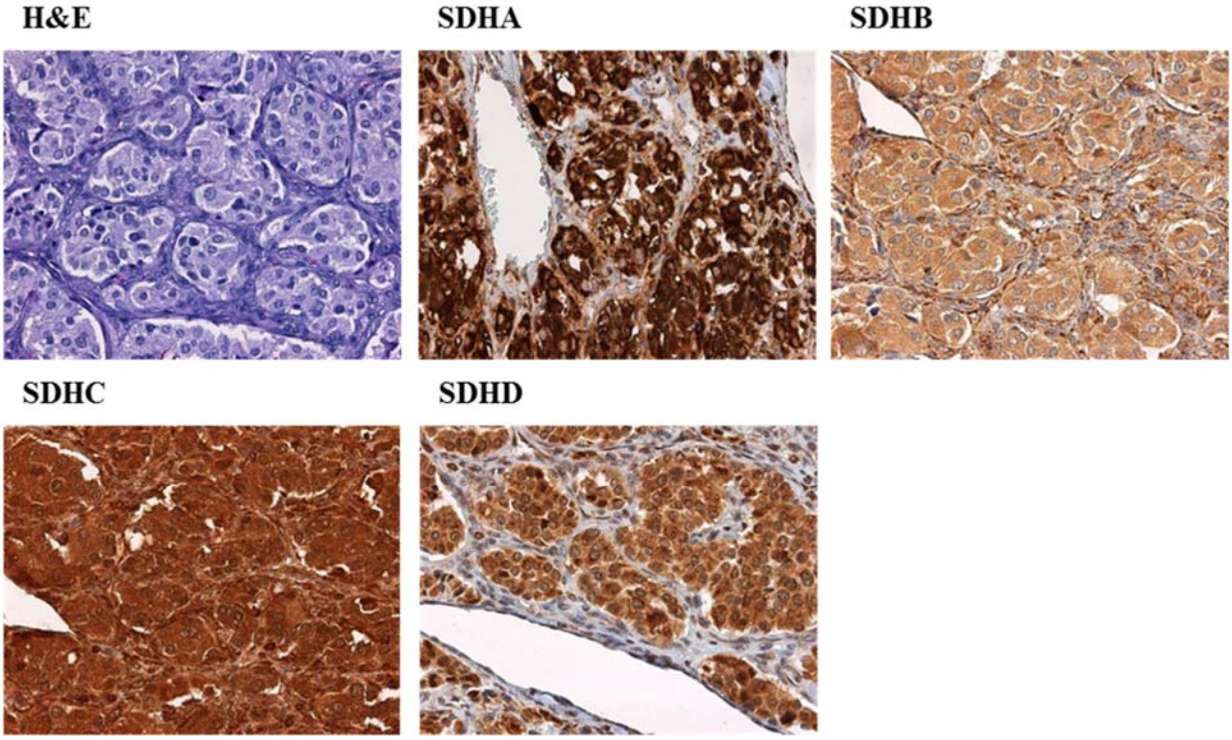

Pat29

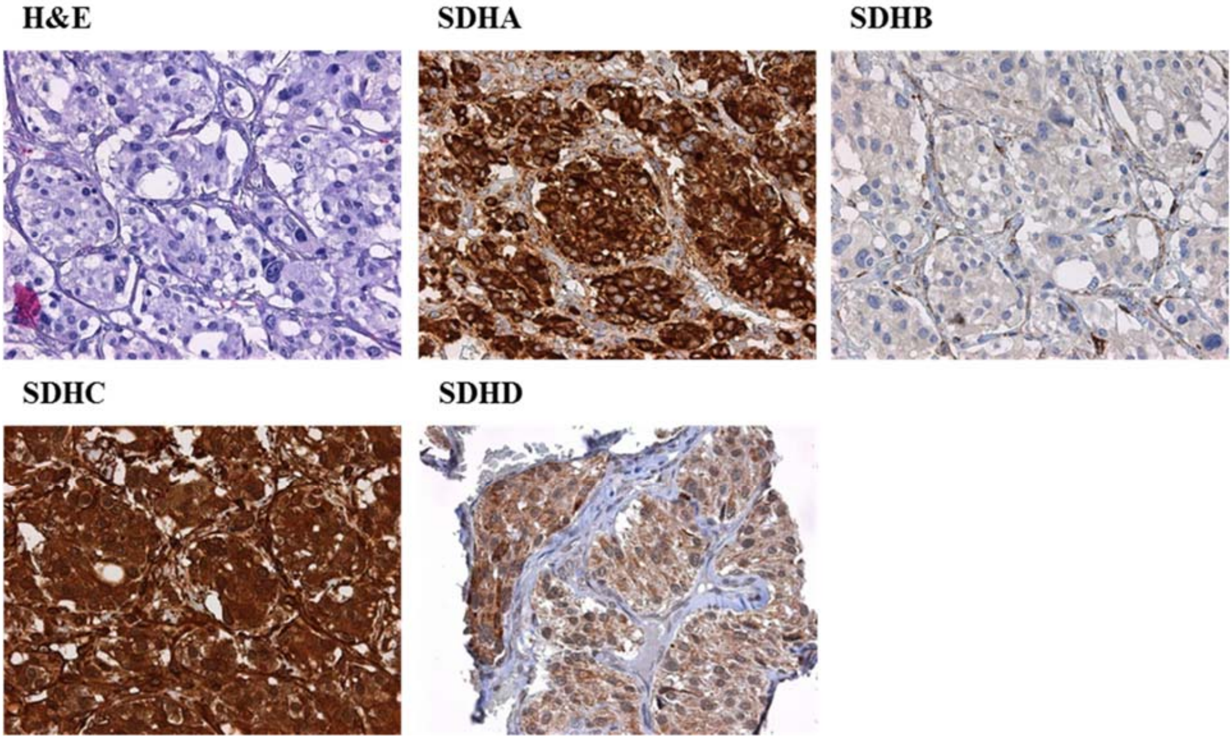

Pat30

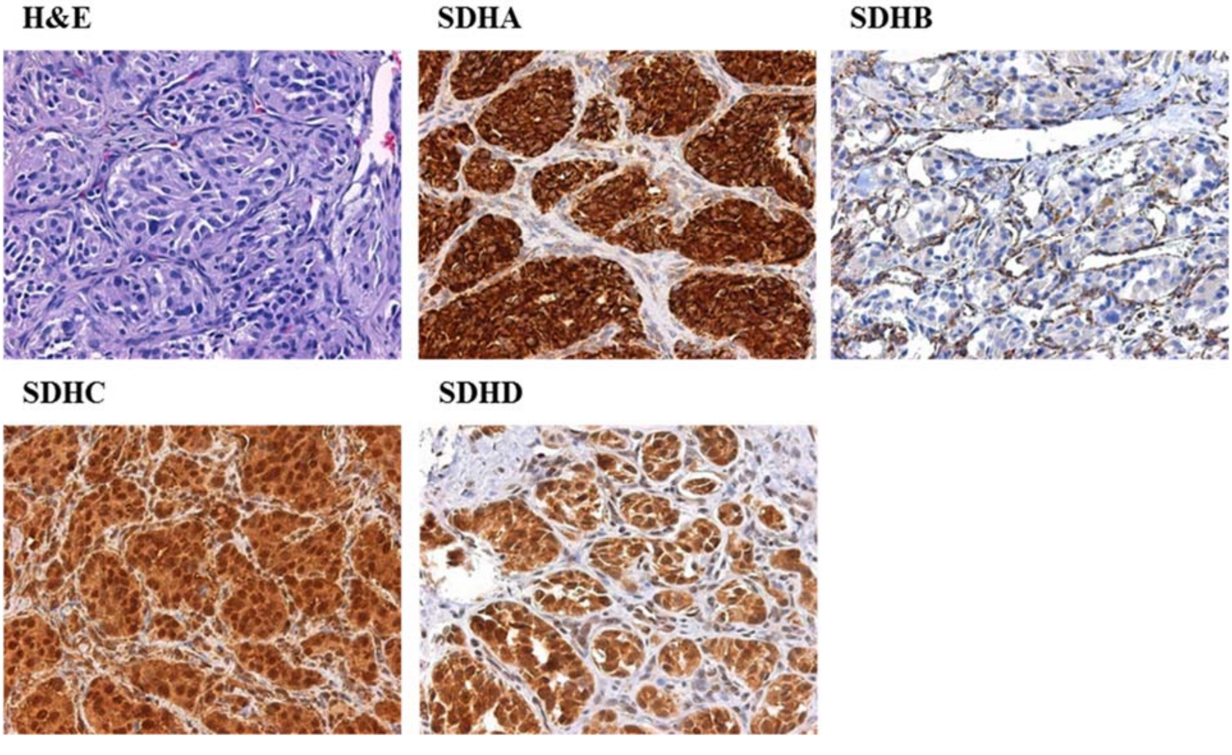

Pat31

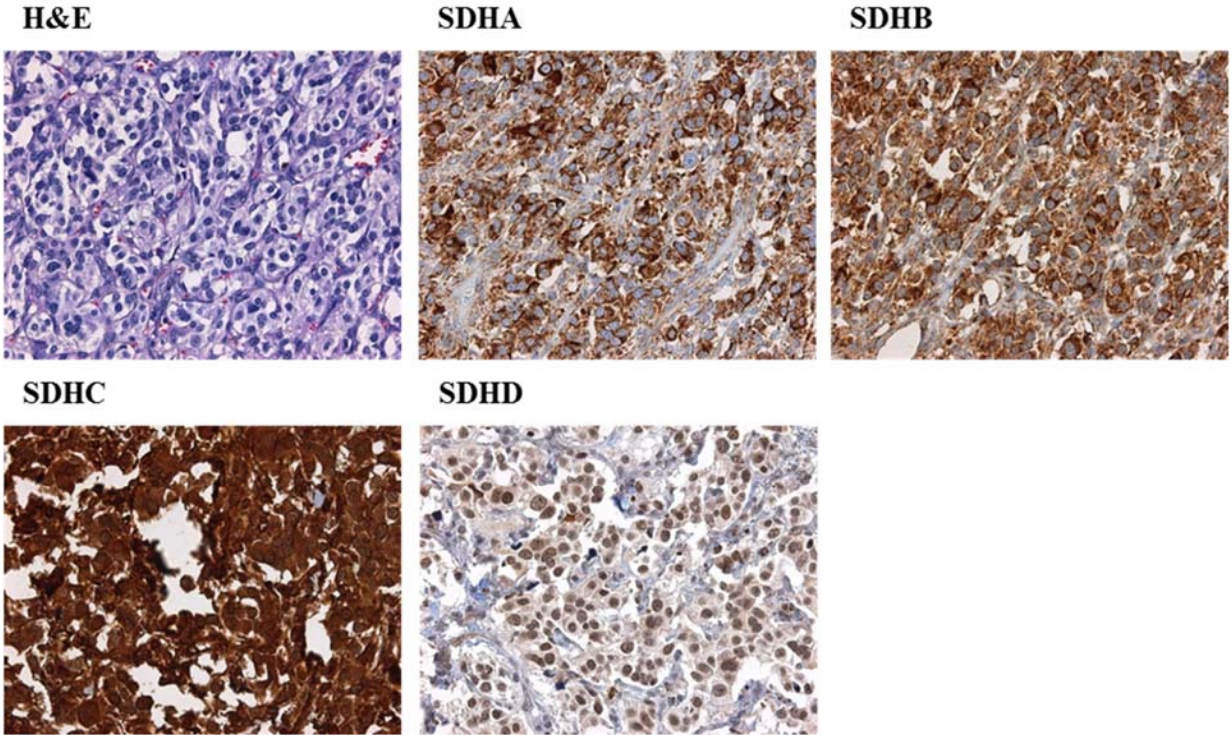

Pat32

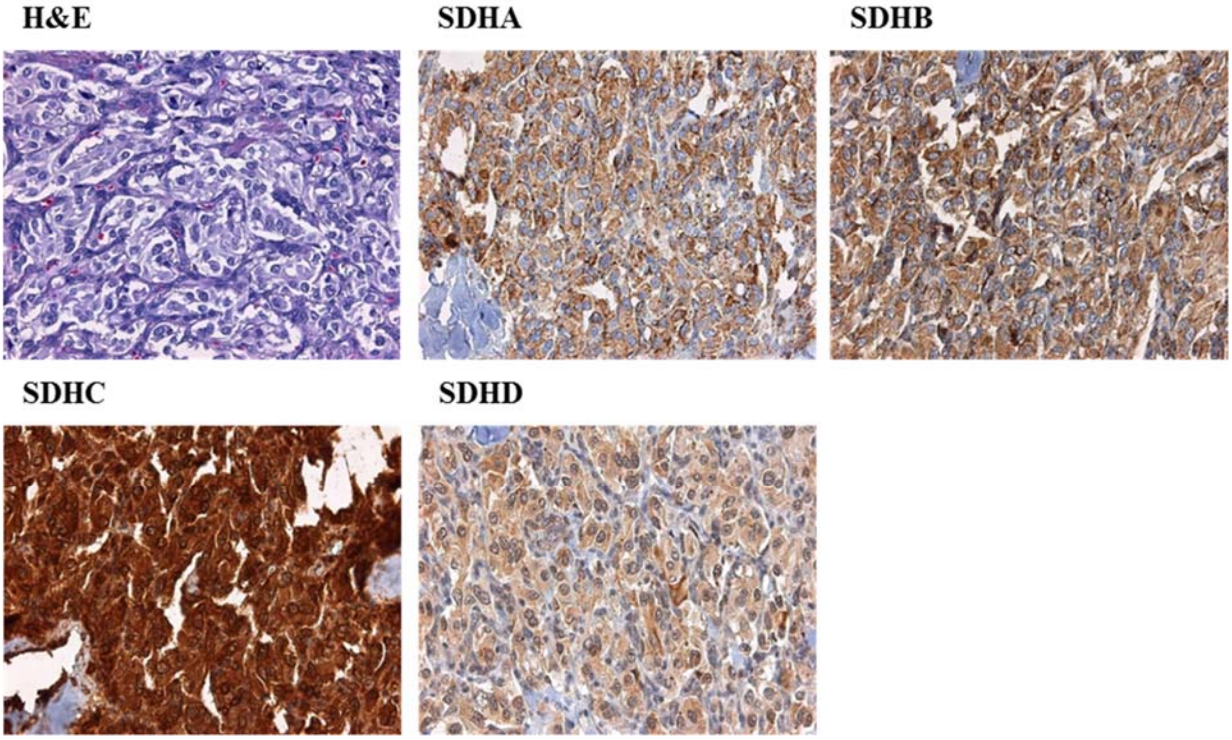

Pat33

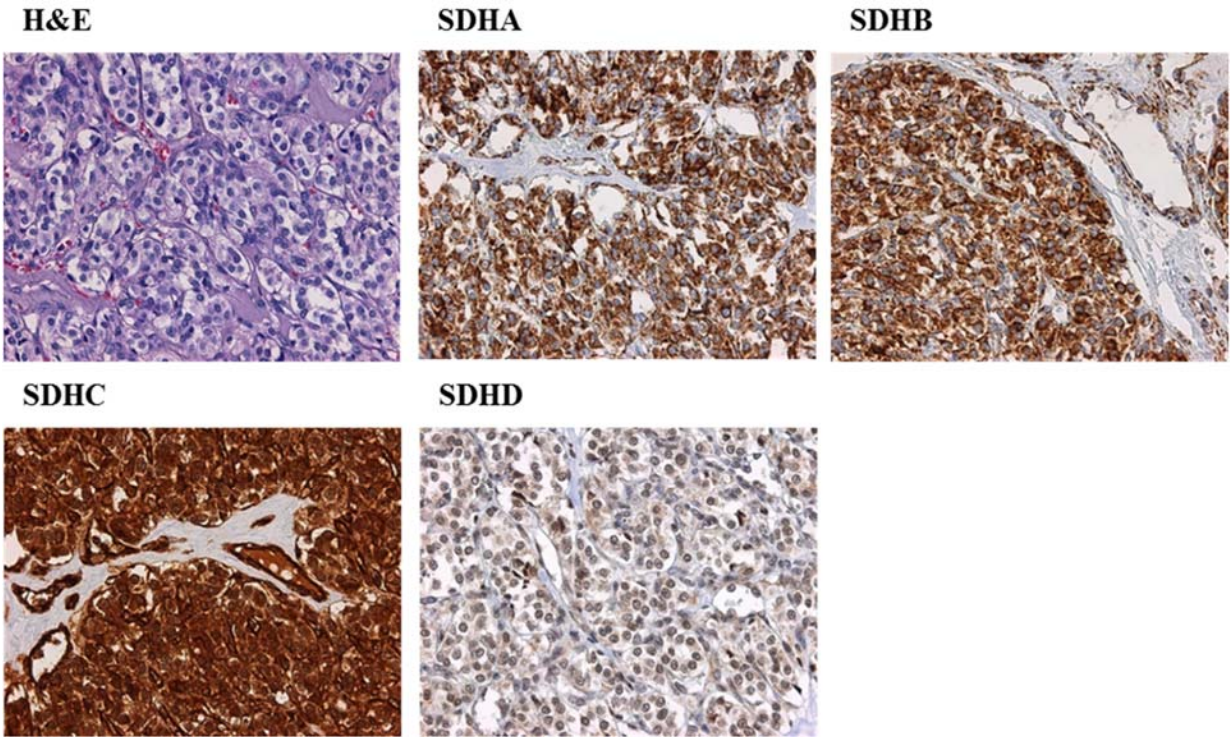

Pat35

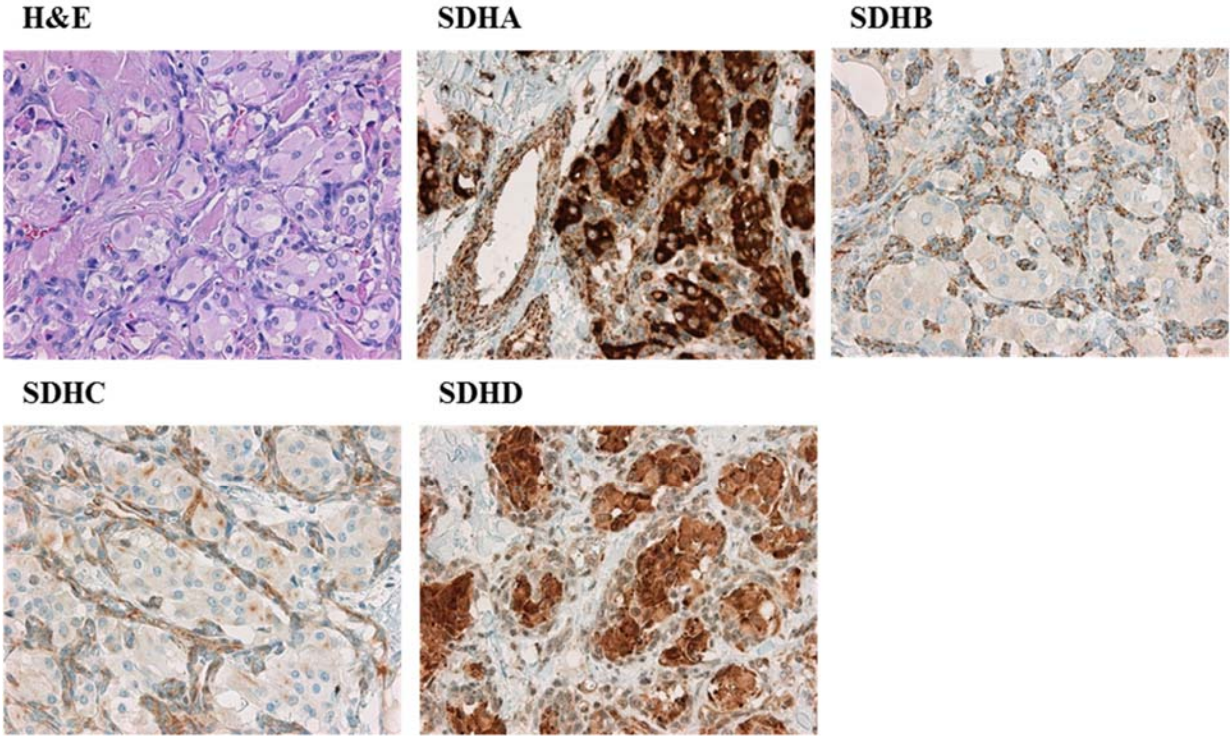

Pat36

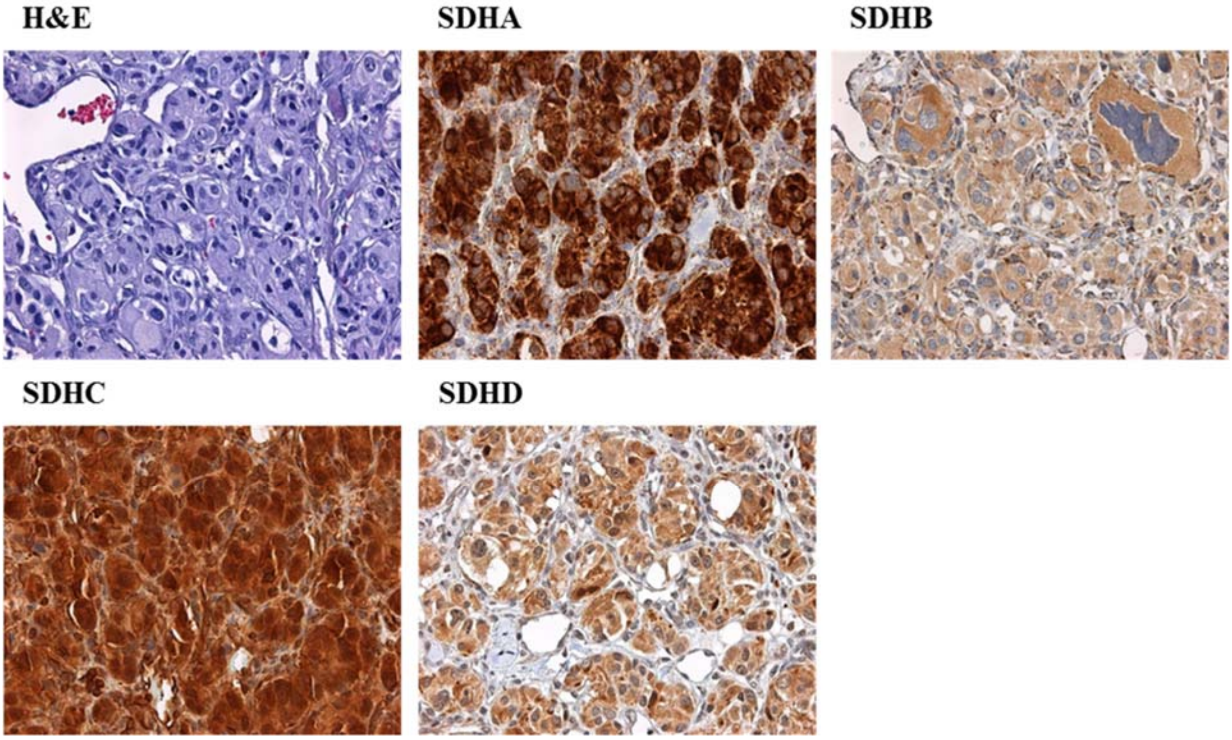

Pat37

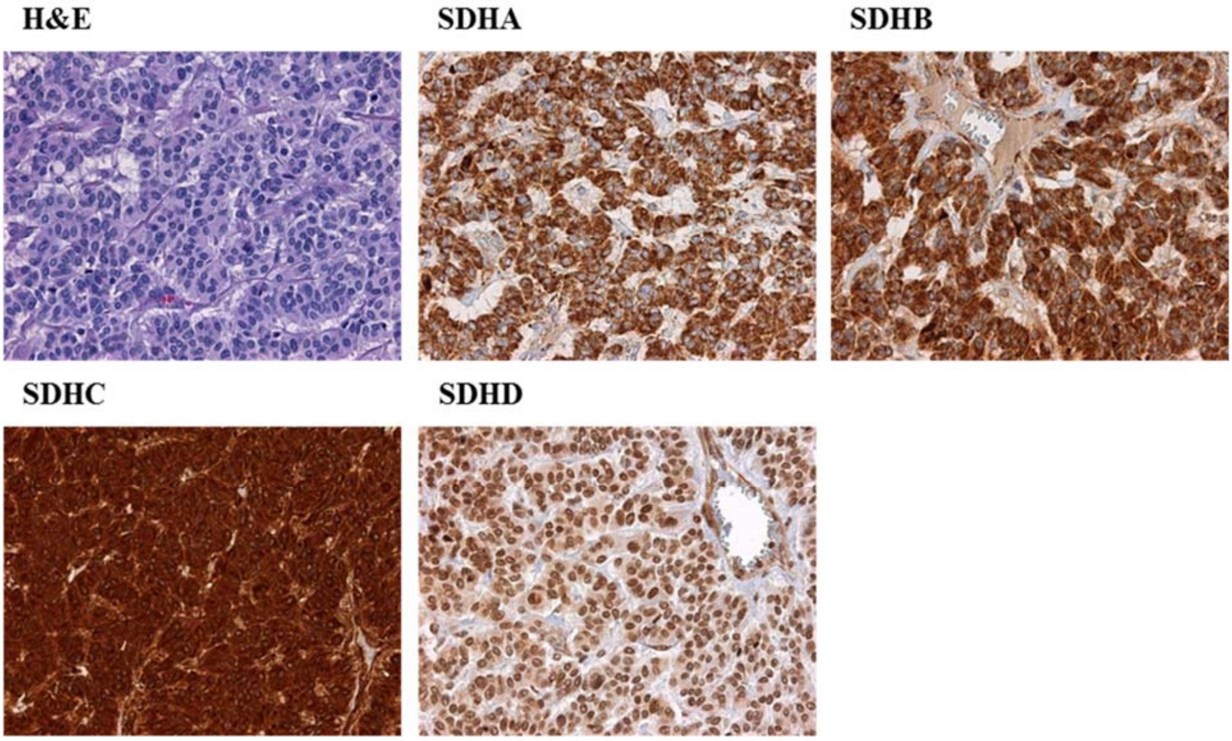

Pat41

H&E

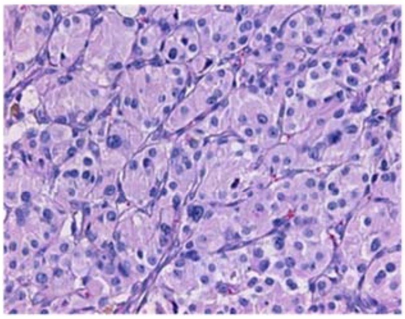

SDHA

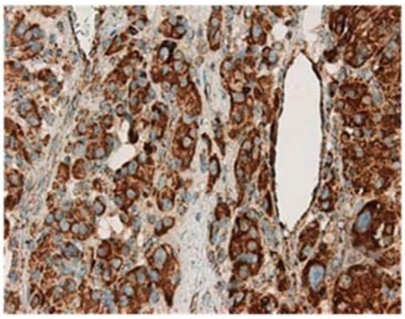

SDHB

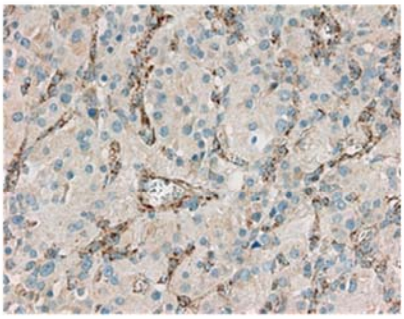

SDHC

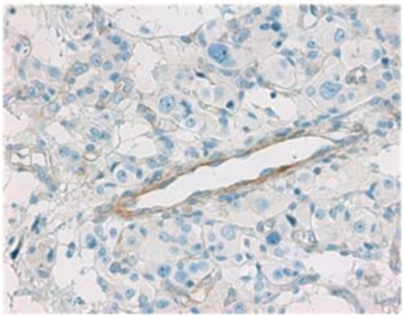

SDHD

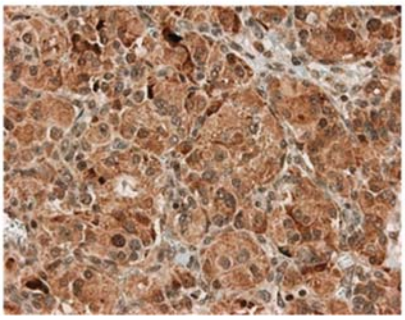

Pat42

H&E

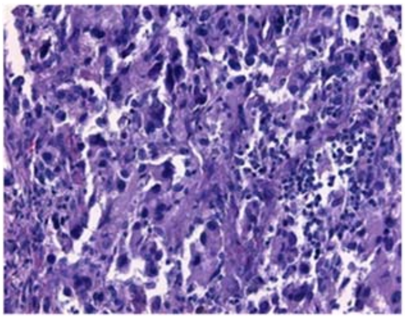

SDHA

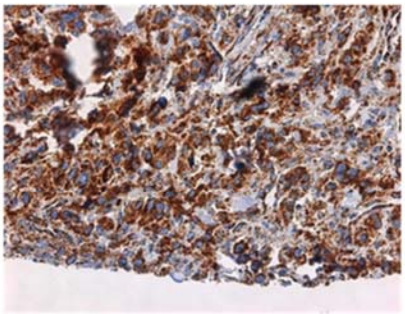

SDHB

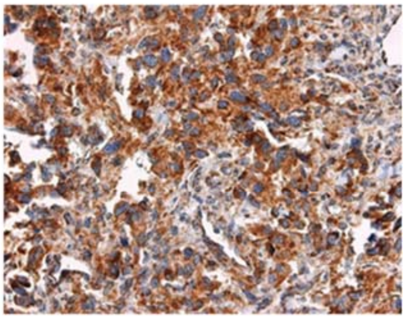

SDHC

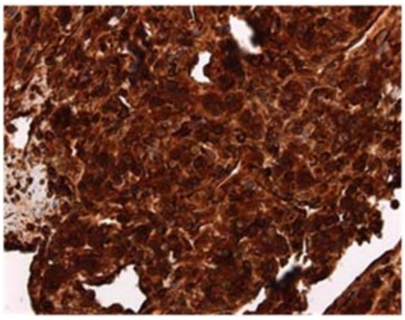

SDHD

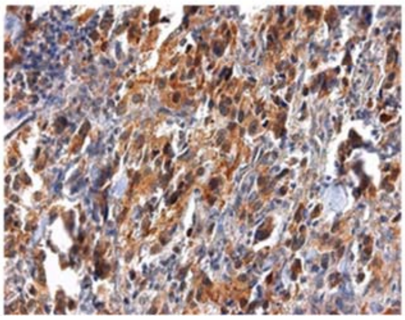

Pat43

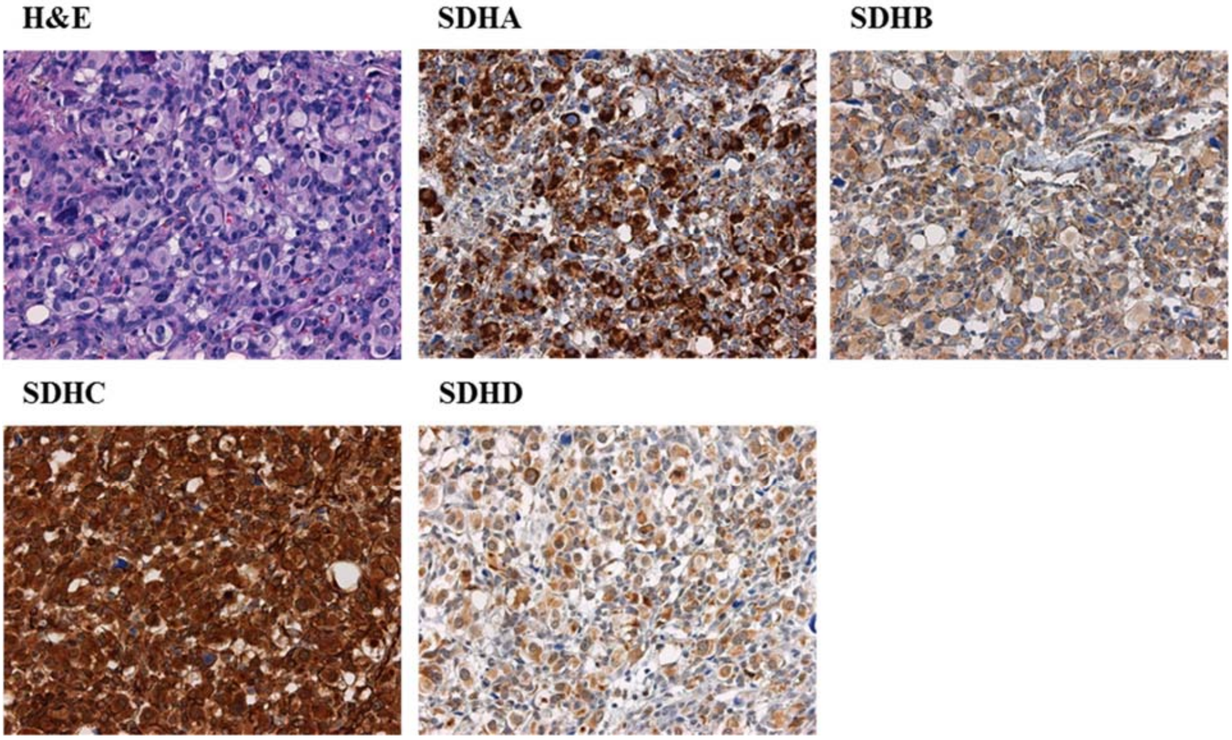

Pat46

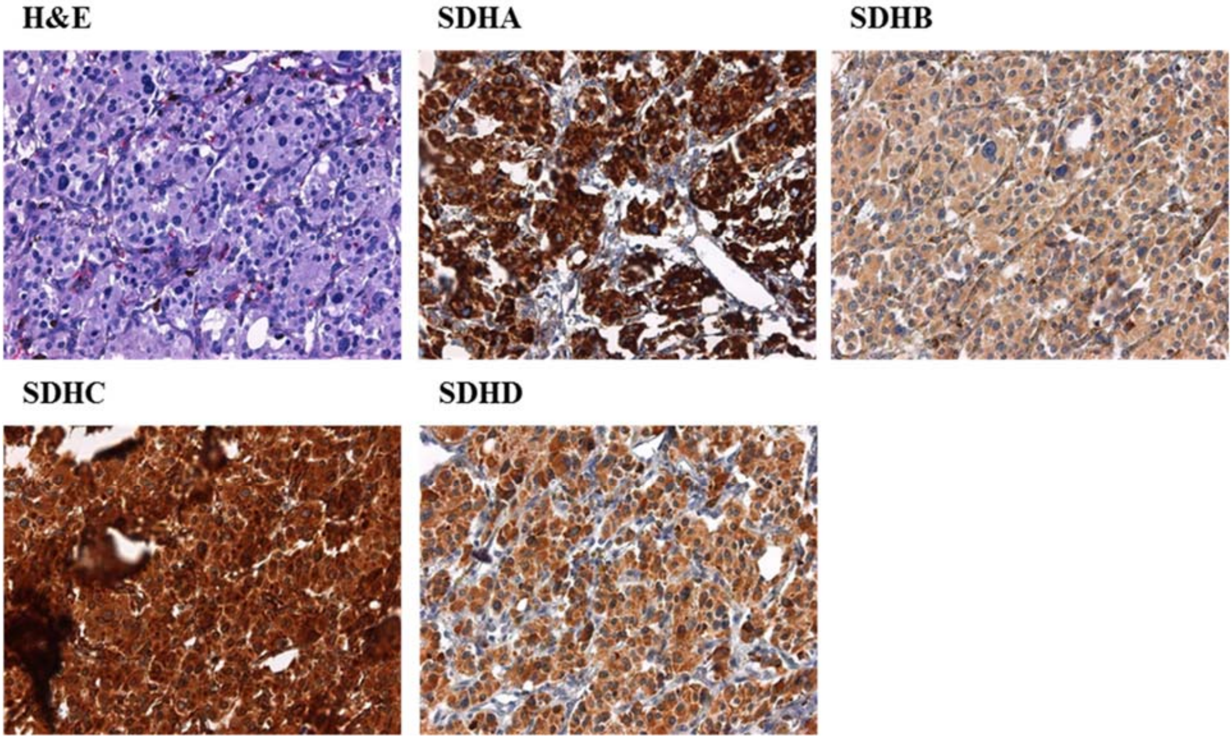

Pat48

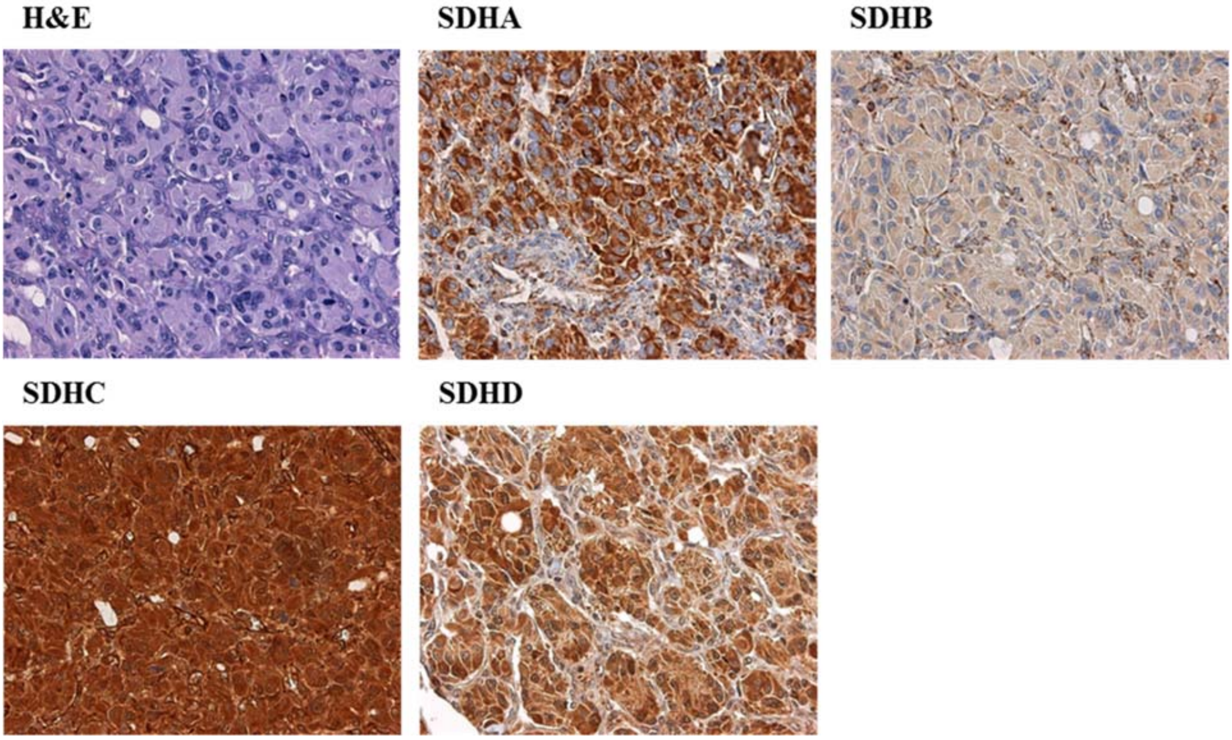

Pat51

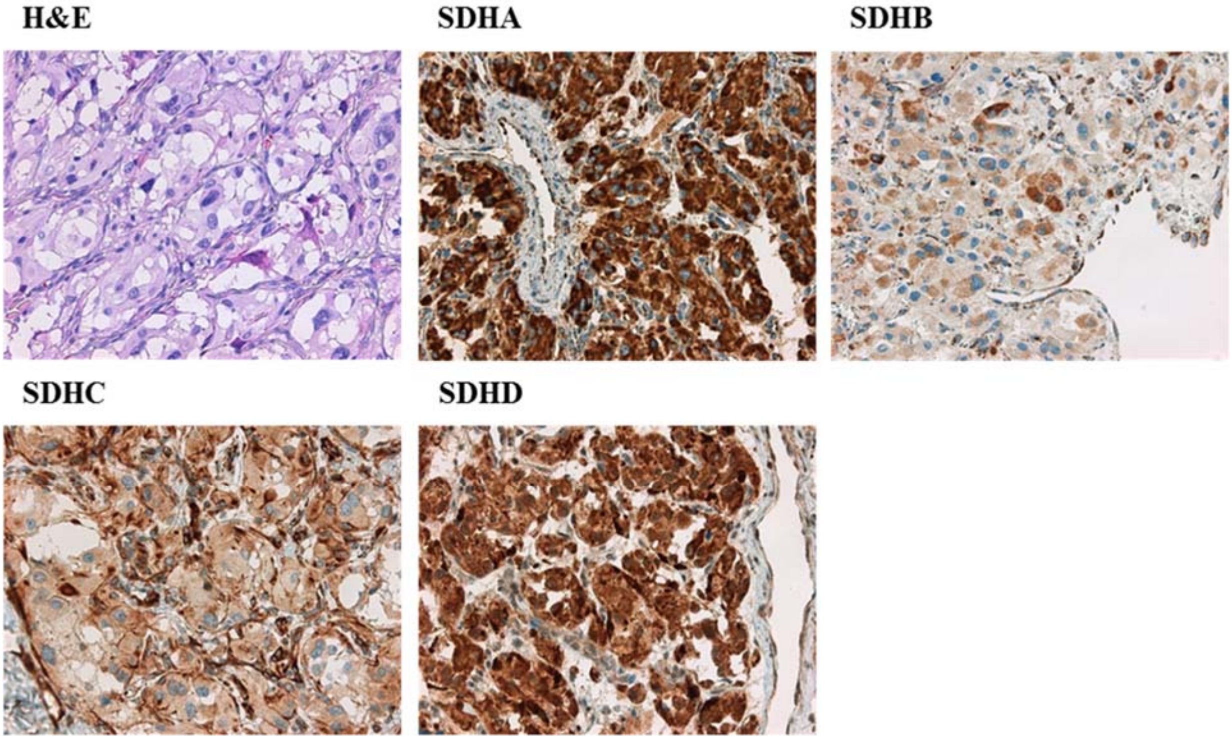

Pat53

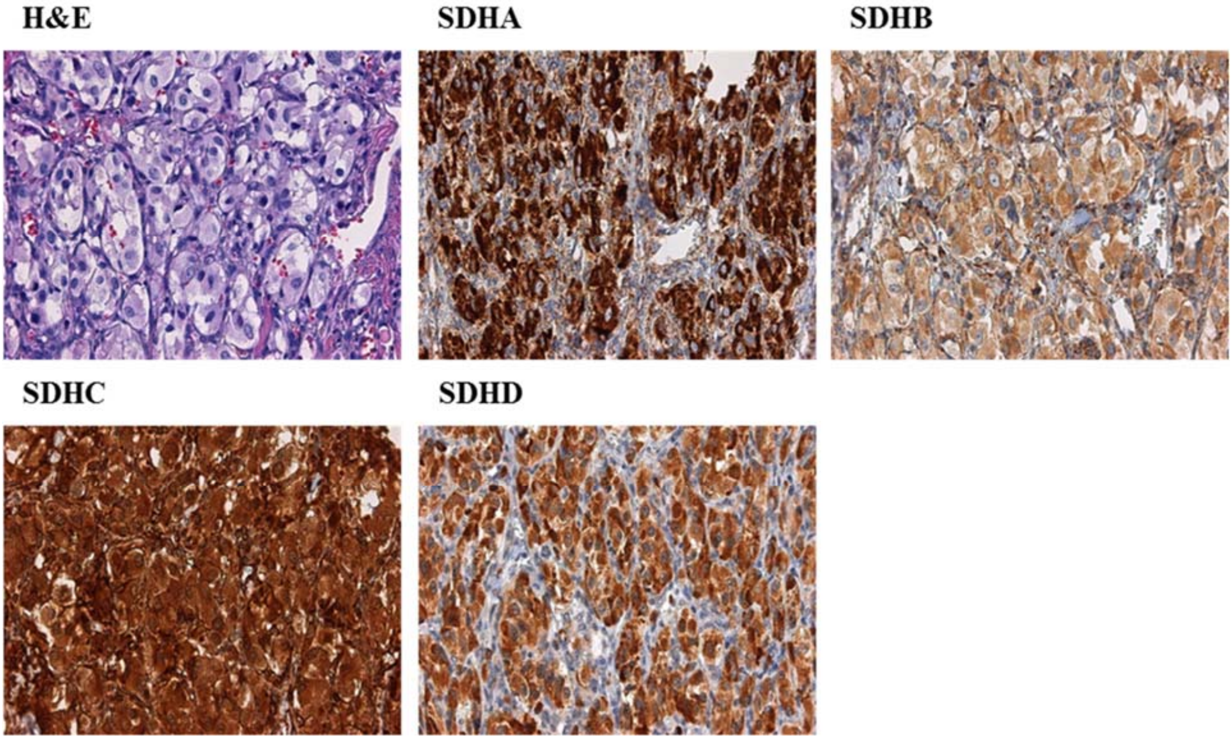

Pat54

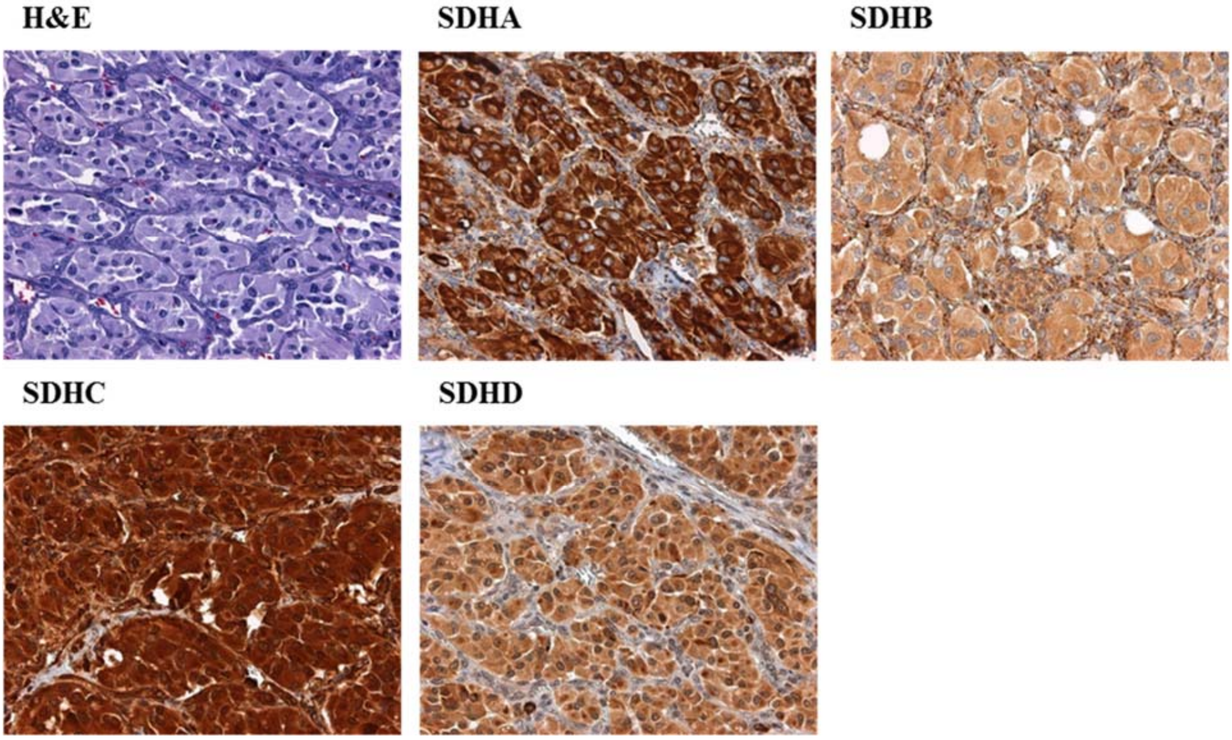

Pat55

H&E

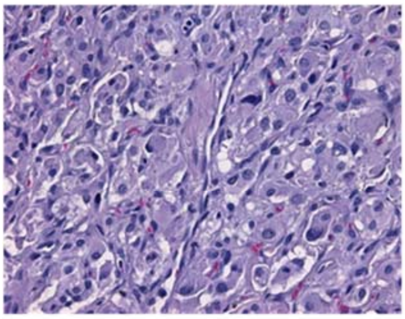

SDHA

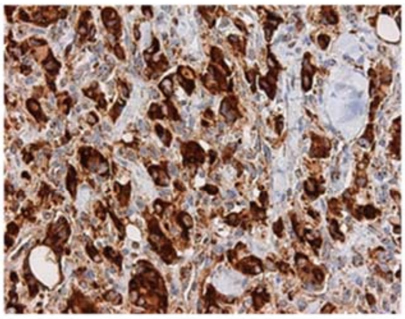

SDHB

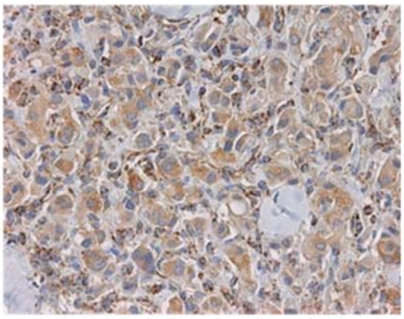

SDHC

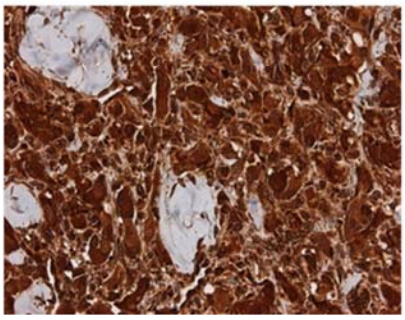

SDHD

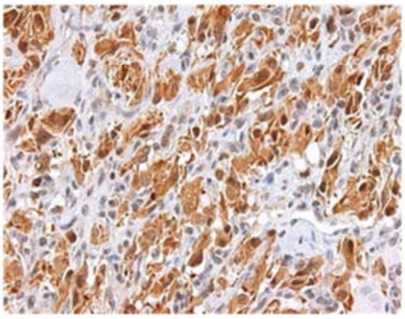

Pat57

H&E

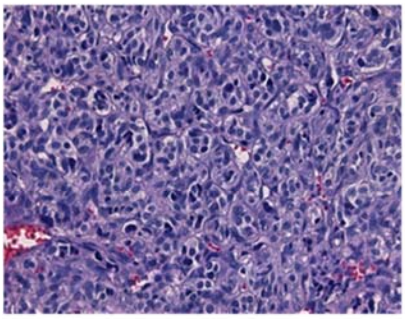

SDHA

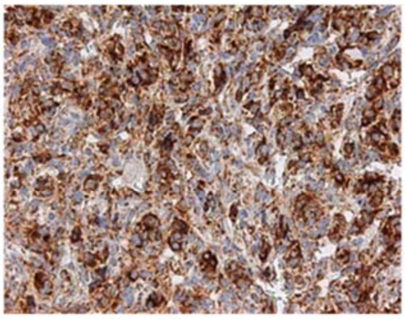

SDHB

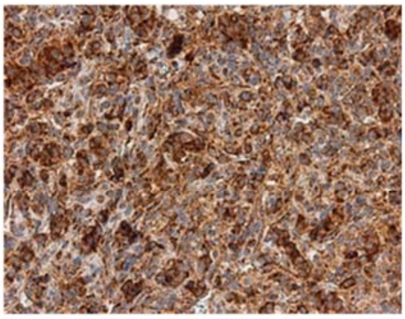

SDHC

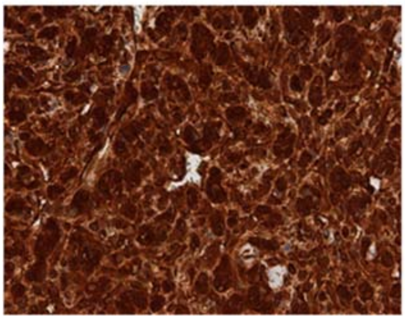

SDHD

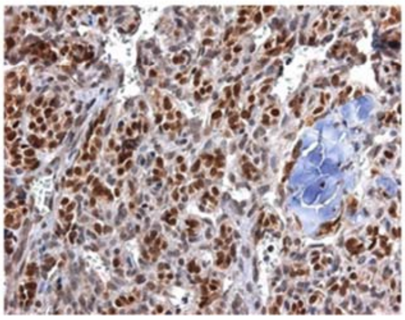

Pat59

H&E

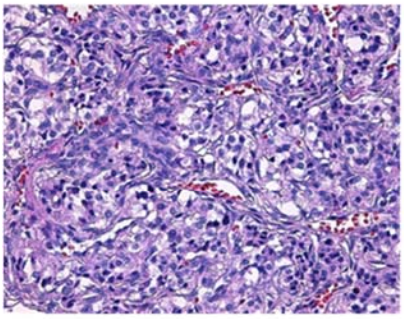

SDHA

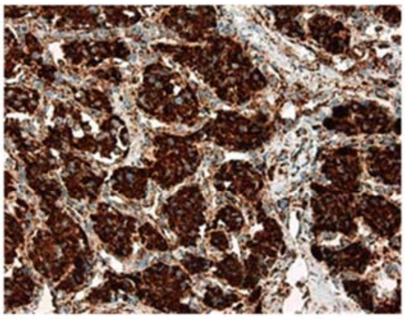

SDHB

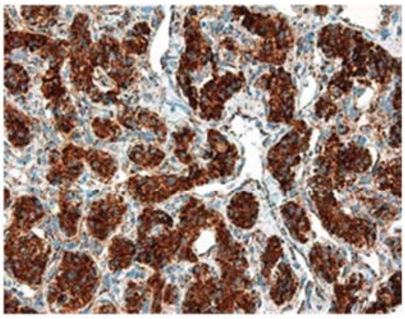

SDHC

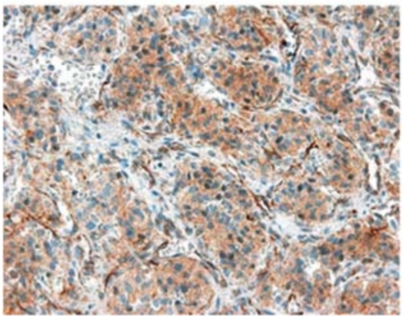

SDHD

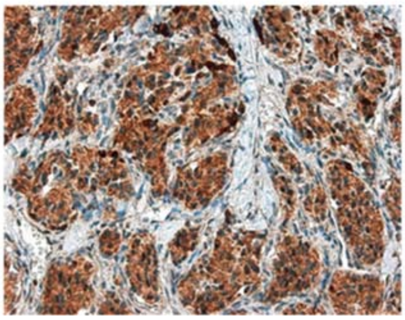

Pat69

H&E

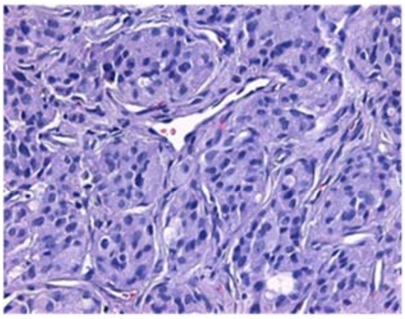

SDHA

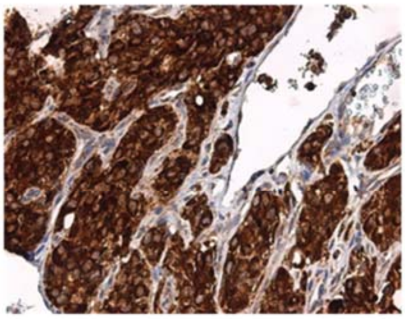

SDHB

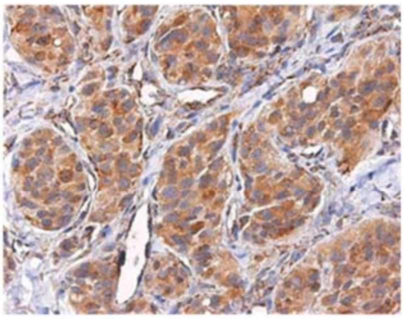

SDHC

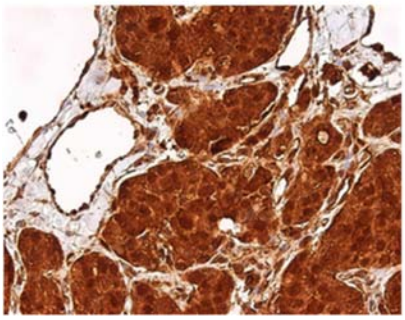

SDHD

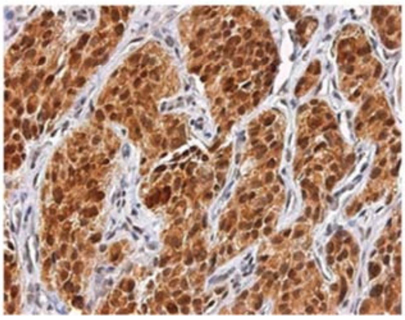

Pat71

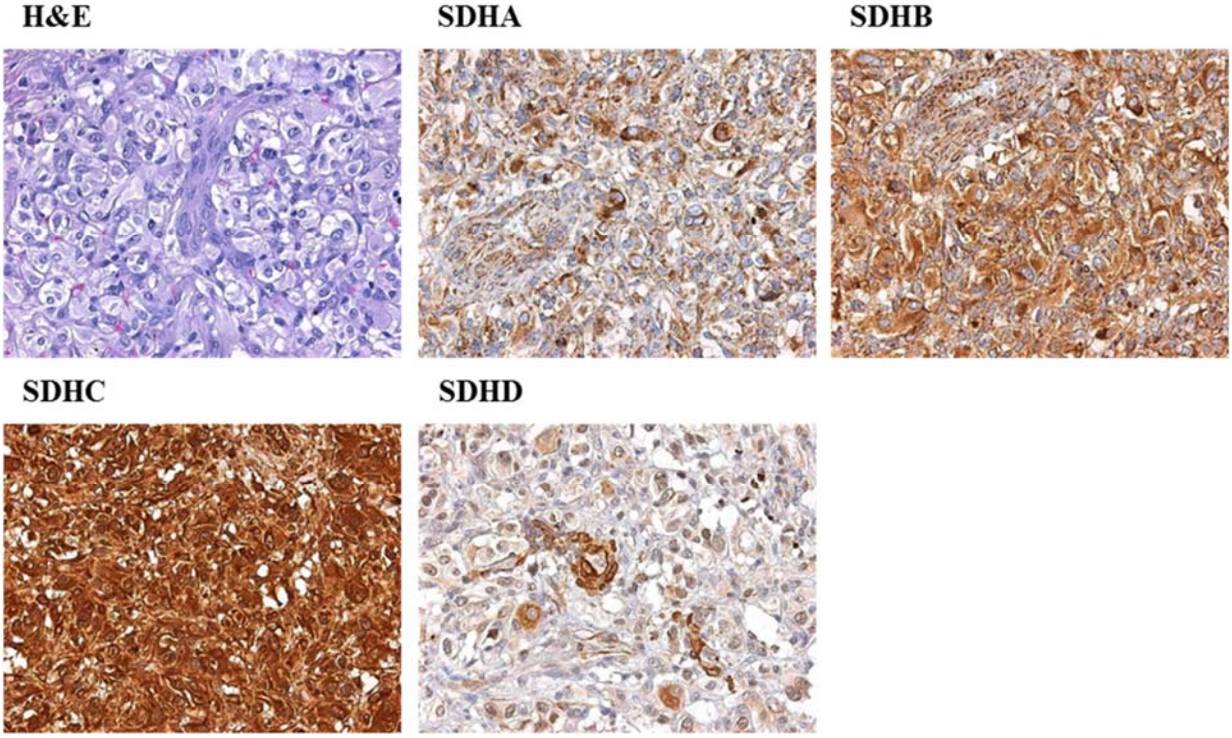

Pat100

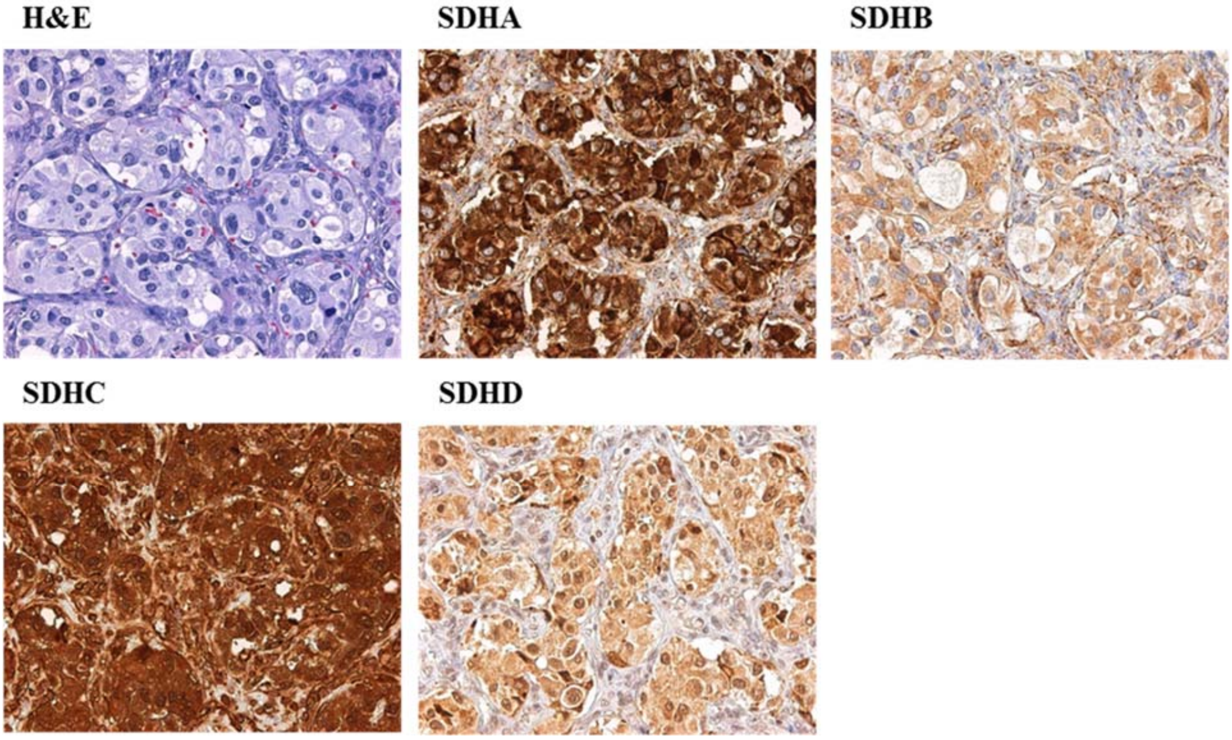

Pat101

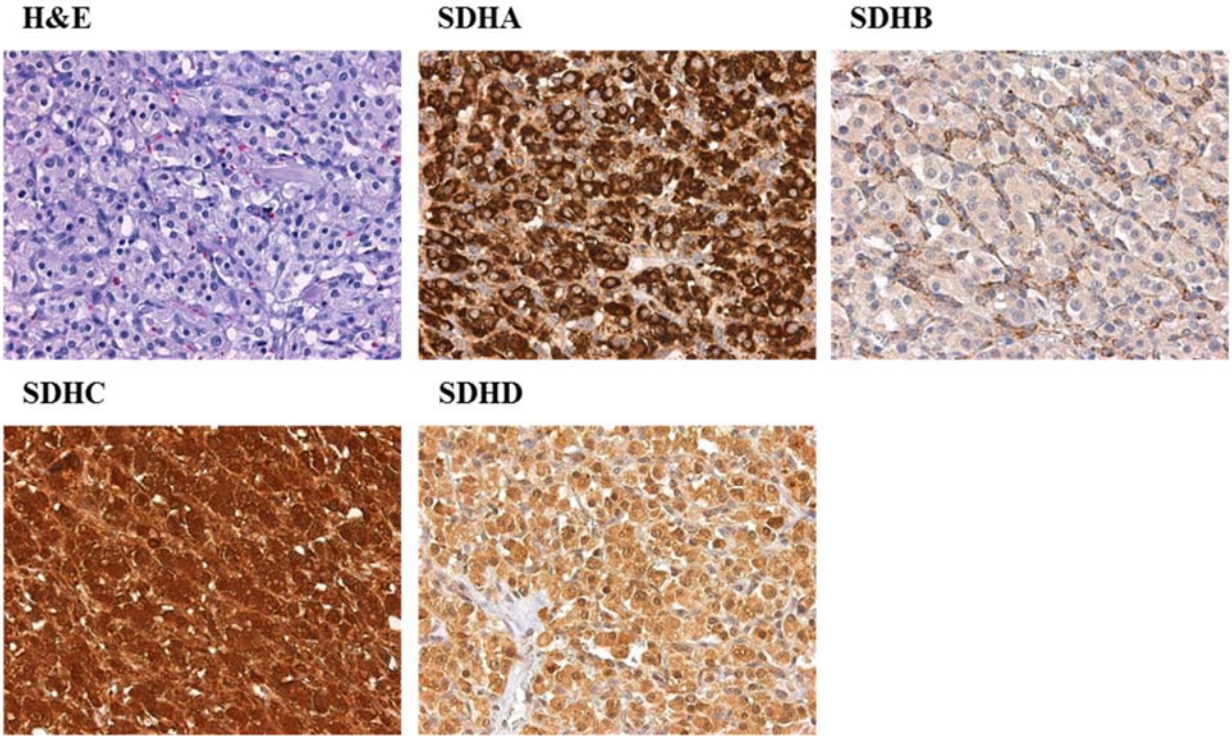

Pat104

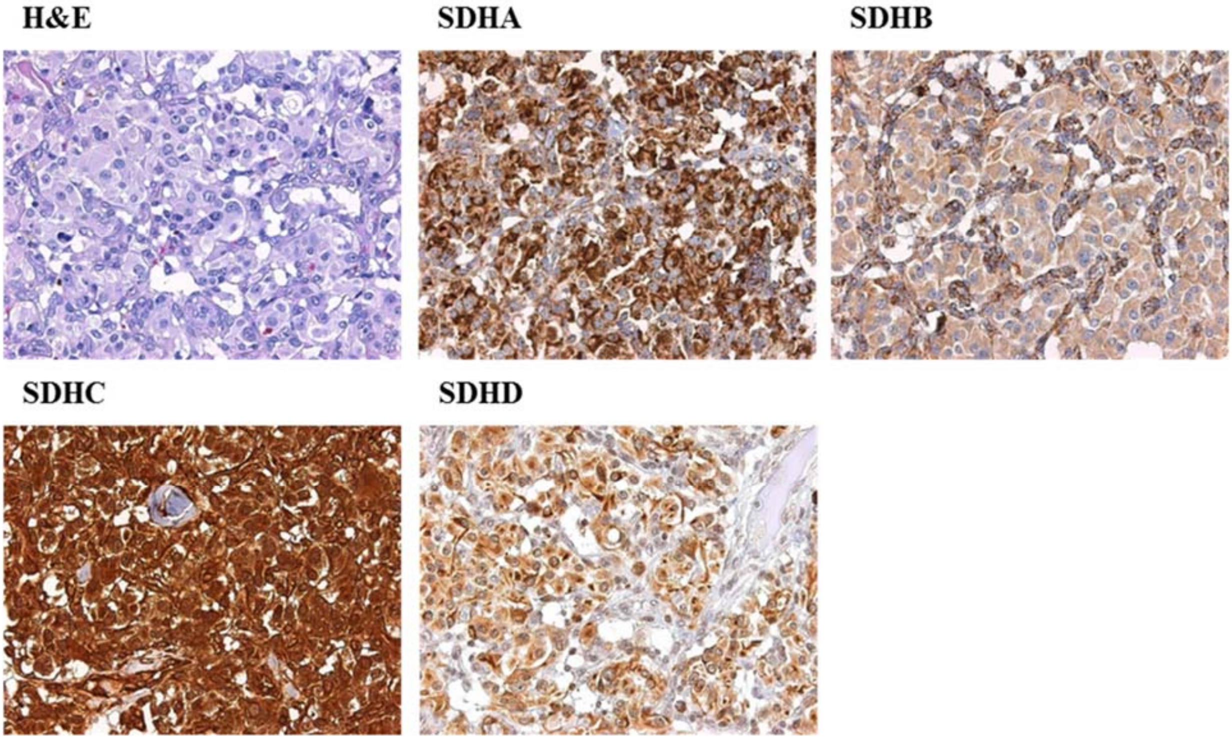

Supplement: Supplementary file 1 [file ijms-21-06950-s001.zip › Supplementary File S1.pdf]
