# Supplementary material for: Immunohistochemistry and Mutation Analysis of SDHx Genes in Carotid Paragangliomas
Source: Int J Mol Sci. 2020 Sep 22;21(18):6950. doi: 10.3390/ijms21186950 (PMC7576476; doi:10.3390/ijms21186950)
Supplement: Supplementary file 1 [file ijms-21-06950-s001.zip › Supplementary Table S1.pdf]

Table S1. Correlation between *SDHx* variants and immunohistochemistry results.

Patient	*SDHx* gene mutated	Immunohistochemistry				Pathogenic/likely pathogenic mutations in other genes tested
		SDHA	SDHB	SDHC	SDHD	
Pat02	*SDHD*	+	*	+	+	
Pat03	*SDHD*	+	*	+	+	
Pat04	No	+	+	+	+	
Pat05	*SDHD*	+	-	+	+	
Pat06	*SDHB*	+	-	+	+	
Pat07	*SDHD*	+	*	+	+	
Pat08	No	+	*	+	+	
Pat09	No	+	+	+	+	
Pat10	*SDHC*	+	*	+	+	
Pat12	No	+	+	+	+	
Pat14	No	+	+	+	+	
Pat16	*SDHA*	-	*	+	+	*RET*
Pat19	No	+	*	+	+	
Pat20	No	+	*	+	+	
Pat22	*SDHD*	+	*	+	+	
Pat23	No	+	+	+	+	
Pat25	No	+	+	+	+	
Pat27	*SDHC*	+	*	+	+	*RET*
Pat29	No	+	-	+	+	
Pat30	No	+	-	+	+	
Pat31	No	+	+	+	+	*IDH1*

Pat32	No	+	+	+	+	
Pat33	No	+	+	+	+	
Pat35	*SDHD*	+	-	+	+	*RET*
Pat36	No	+	*	+	+	
Pat37	No	+	+	+	+	
Pat41	*SDHC*	+	-	-	+	
Pat42	No	+	+	+	+	
Pat43	No	+	+	+	+	
Pat46	No	+	*	+	+	
Pat48	No	+	*	+	+	
Pat51	No	+	*	+	+	
Pat53	No	+	*	+	+	
Pat54	No	+	*	+	+	
Pat55	*SDHD*	+	*	+	+	
Pat57	No	+	+	+	+	
Pat59	No	+	+	+	+	
Pat69	*SDHD*	+	+	+	+	
Pat71	No	+	+	+	+	
Pat100	*SDHD*	+	*	+	+	
Pat101	*SDHB*	+	-	+	+	
Pat104	*SDHD*	+	*	+	+	

(+) - positive staining; (-) – negative staining; (*) - weak diffuse staining.
