# Supplementary figures and images for: Immunohistochemistry and Mutation Analysis of SDHx Genes in Carotid Paragangliomas
Source: Int J Mol Sci. 2020 Sep 22;21(18):6950. doi: 10.3390/ijms21186950 (PMC7576476; doi:10.3390/ijms21186950)

**SDHA**

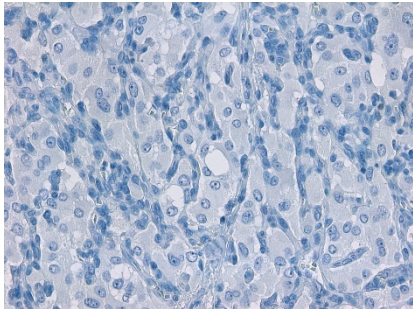

**SDHB**

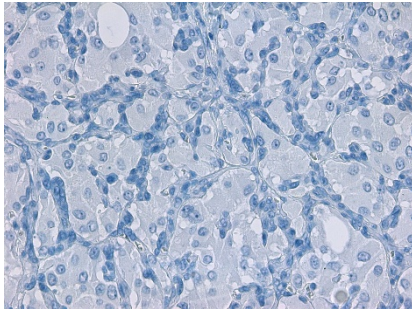

**SDHC**

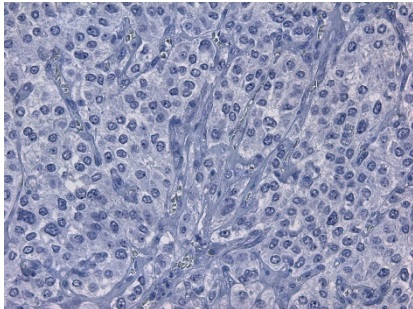

**SDHD**

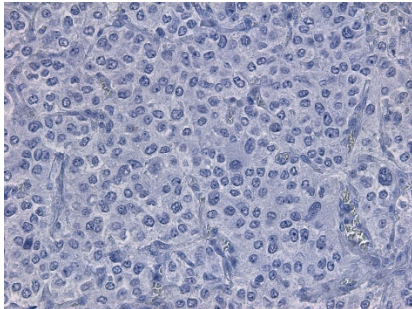

Supplement: Supplementary file 1 [file ijms-21-06950-s001.zip › Supplementary Figure S1.pdf]
